# Quantitative Design and Residual Strength Assessment of Adhesive–Rivet Hybrid Repairs for Perforated Aluminum Alloy Plates

**DOI:** 10.3390/polym18172126

**Published:** 2026-08-31

**Authors:** Antai Ren, Teng Zhang, Tao An, Liying Ma

**Affiliations:** Aviation Engineering School, Air Force Engineering University, Xi’an 710043, China

**Keywords:** adhesive–rivet hybrid repair, quantitative repair design, residual strength assessment, cohesive zone model, perforated aluminum alloy plates

## Abstract

Perforation damage can significantly reduce the load-carrying capacity of aluminum alloy plates. Adhesive–rivet hybrid repair combines the continuous load-transfer capability of adhesive bonding with the reliable mechanical connection provided by riveting; however, quantitative methods for matching damage size, rivet parameters, and adhesive load-carrying capacity remain insufficient. In this study, perforated 2A12-T4 aluminum alloy plates were investigated. Based on the equal-strength criterion and load-transfer equilibrium, a strength-matching relationship between the adhesive layer and blind rivets was established, and a residual-strength assessment method for the repaired structure was proposed. Two typical two-part epoxy adhesives with different shear strengths, Araldite-2015 and Lord 320/322, which have application backgrounds in aerospace structural joining and repair, were selected. Combined with blind rivets of different load-carrying capacities, they formed strong-adhesive/weak-rivet and weak-adhesive/strong-rivet configurations to investigate the mechanical response under different adhesive–rivet strength-matching conditions. Quasi-static tensile tests, digital image correlation (DIC) measurements, and finite element analyses incorporating a cohesive zone model and a ductile damage criterion were performed to investigate load distribution and failure behavior. The results show that the maximum deviation between the finite element predictions and the experimental failure loads is 5.02%. Before significant adhesive failure, the adhesive layer carries up to 67.25% of the transferred load, indicating a substantial load-sharing effect on the rivets. The hybrid-repaired structures mainly fail along the cross-section through the outermost rivet holes. The proposed residual-strength model shows agreement with the investigated experimental dataset, with a maximum deviation of 6.37%; because the reduction coefficient contains an empirical calibration component, broader predictive applicability requires independent validation. For the six repair configurations, the strength recovery ratios all exceed 74%, the maximum strengthening ratio reaches 55.49%, and the maximum value of the newly proposed repair ratio is 0.48 kN/g. Unlike previous studies that mainly focused on comparisons of joining methods, failure behavior, or individual process parameters, this study establishes a quantitative framework that links damage size and material load-carrying capacity with adhesive–rivet parameter matching and post-repair residual-strength assessment. The proposed method provides theoretical and experimental support for the design and strength evaluation of adhesive–rivet hybrid repairs for perforated aluminum alloy thin plates under fully cured conditions.

## 1. Introduction

Aluminum alloy thin-walled structures are widely used in lightweight load-bearing components, particularly in aerospace structures, owing to their high specific strength and good manufacturability. When such structures are subjected to impact, local damage, or other external actions that result in perforation damage, the effective load-bearing cross-section is reduced and pronounced stress concentration develops around the hole, leading to a decrease in residual strength. Therefore, an appropriate repair method is required to restore the load-transfer capability of the damaged structure. Mechanical riveting offers reliable connection performance and mature manufacturing technology; however, rivet holes introduce additional local stress concentrations. Structural adhesive bonding, in contrast, enables continuous interfacial load transfer and promotes a more uniform stress distribution within the joint region, although its performance is influenced by adhesive properties, bondline thickness, surface treatment, and curing conditions [1]. By combining adhesive bonding with mechanical fastening, adhesive–rivet hybrid joints can exploit the complementary load-bearing mechanisms of the two joining methods and have therefore become an important research direction for the joining and repair of metallic thin-walled structures [2].

Accurate characterization of adhesive properties and interfacial damage is essential for analyzing load transfer in hybrid joints. In recent years, relevant research has gradually progressed from the evaluation of single-material strength to the investigation of mechanical responses under environmental effects, loading-rate variations, and mixed-mode fracture conditions. Schmelzle et al. [3] quantified the coupled effects of temperature, ranging from −40 °C to 60 °C, and loading rate on epoxy adhesives. Their results showed that low temperatures and high loading rates can significantly increase the yield strength and modulus of the adhesive, while potentially reducing its toughness. Han et al. [4] investigated adhesively bonded aluminum alloy joints under hygrothermal aging conditions and developed a new constitutive model by incorporating moisture-induced plasticization and cyclic-loading-induced stiffness degradation to predict fatigue damage evolution. The cohesive zone model (CZM), which describes damage initiation and progressive propagation in the adhesive layer through a traction–separation relationship, has been widely applied to the numerical simulation of adhesively bonded and adhesive–mechanical hybrid joints. Refined identification of cohesive parameters and the incorporation of complex environmental effects have substantially improved the accuracy of numerical predictions. Yahya et al. [5], based on a bilinear CZM, demonstrated that accurate determination of the critical strain energy release rate and interfacial strength is essential for reliable prediction. Hasumi et al. [6] developed a nonlinear power-law mixed-mode fracture criterion, which was shown to reproduce the fracture behavior of CFRP/aluminum alloy joints more accurately than a linear superposition model.

In engineering applications, the load-bearing mechanisms and parameter effects of hybrid joints combining adhesive bonding and mechanical fastening have been investigated relatively systematically in recent years. Regarding post-repair strength, Pitta et al. [7] investigated the mechanical performance and failure mechanisms of mechanically fastened (riveted), adhesively bonded, and hybrid joints applied to composite structures. They found that hybrid joints integrate the characteristics of mechanical fastening and adhesive bonding and can effectively suppress the initiation and propagation of defects and damage. Zhang et al. [8] compared the fatigue performance of perforated metallic skin panels repaired by riveting, adhesive bonding, and adhesive–rivet hybrid techniques through experimental tests and finite element analysis. Their results showed that the adhesive–rivet hybrid repair provided the best repair performance, with a fatigue life 2.396 times that of the unrepaired perforated plate, while effectively alleviating stress concentrations, enhancing load transfer through the adhesive layer, and suppressing adhesive debonding. With respect to process-parameter optimization, Yokozeki et al. [9] investigated hybrid joints combining pretensioned bolts with adhesive bonding and demonstrated that adhesive type directly affects the load-bearing capacity. High-modulus adhesives were found to improve the initial stiffness; whereas, tougher adhesives were more beneficial for energy absorption. Yan et al. [10] studied self-piercing riveted (SPR)–adhesive hybrid joints and identified riveting pressure, aluminum-sheet surface treatment, and overlap length as key parameters governing mechanical performance. Su et al. [11] employed regression-model-based optimization to determine an optimal combination of geometric parameters for CFRP–aluminum alloy hybrid joints, achieving a balance between structural strength and lightweight design.

Although existing studies have systematically investigated the mechanical behavior, failure mechanisms, and parameter optimization of adhesive–rivet hybrid joints, two major limitations remain in the repair of perforated metallic plates. First, a quantitative relationship among damage-hole size, rivet configuration, and adhesive load-carrying capacity has not yet been established, making it difficult to directly determine repair parameters from the damage state. Second, variations in damage size and rivet layout can alter the critical sections and failure modes, while existing methods remain inadequate for accurately assessing the residual strength of repaired structures. Accordingly, this study focuses on two aspects: (1) establishing a strength-matching method among damage size, rivet parameters, and adhesive shear load-carrying capacity based on the equal-strength and stiffness-coordination principles; and (2) developing a residual-strength assessment model considering multiple failure modes by combining the effective base-plate section, rivet shear capacity, and adhesive load-carrying capacity, and evaluating different repair schemes using the strength recovery ratio, strengthening ratio, and a newly proposed repair ratio.

To address the difficulty in quantitatively determining repair parameters for adhesive–rivet hybrid repairs and the lack of effective methods for assessing post-repair residual strength, this study follows a research framework of “theoretical design–experimental validation–numerical analysis–strength assessment.” First, the load-transfer relationship of the adhesive–rivet hybrid structure is analyzed based on the equal-strength and stiffness-coordination principles, and a strength-matching method is established among rivet geometric parameters, rivet number, and adhesive load-carrying capacity. Second, hybrid-joint segment tests with different adhesive–rivet combinations and perforated-plate repair tests with different damage-hole sizes and rivet configurations are conducted to validate the proposed strength-matching relationship and to investigate the effects of repair parameters on load-carrying capacity and failure modes. Subsequently, a finite element model incorporating adhesive damage and metallic failure is developed to investigate load sharing between the adhesive layer and rivets, damage evolution, and the formation mechanism of critical sections. Finally, a residual-strength assessment model considering multiple failure modes is established based on the combined load-carrying contributions of the base plate, rivets, and adhesive layer, and different repair configurations are evaluated using the strength recovery ratio, strengthening ratio, and repair ratio. The results provide a basis for parameter design and residual-strength assessment of adhesive–rivet hybrid repairs for perforation-damaged aluminum alloy thin plates.

## 2. Hybrid Adhesive–Rivet Repair Scheme

Damaged aircraft structures shall comply with the equal strength criterion and the stiffness compatibility criterion. The equal strength criterion requires the repair patch to compensate for the strength loss of the base plate caused by perforation. The stiffness compatibility criterion requires stiffness matching between the base plate and the patch as well as a compatible load transfer path; accordingly, the patch is typically made of the same material and has the same thickness as the base plate. For riveted repair of damaged panel structures, specific principles must be followed. The rivet material, rivet diameter and rivet distribution shall be subject to detailed verification, so as to determine the final repair scheme and evaluate the final repair effect.

### 2.1. Rivet Material and Diameter

**Figure 1:**
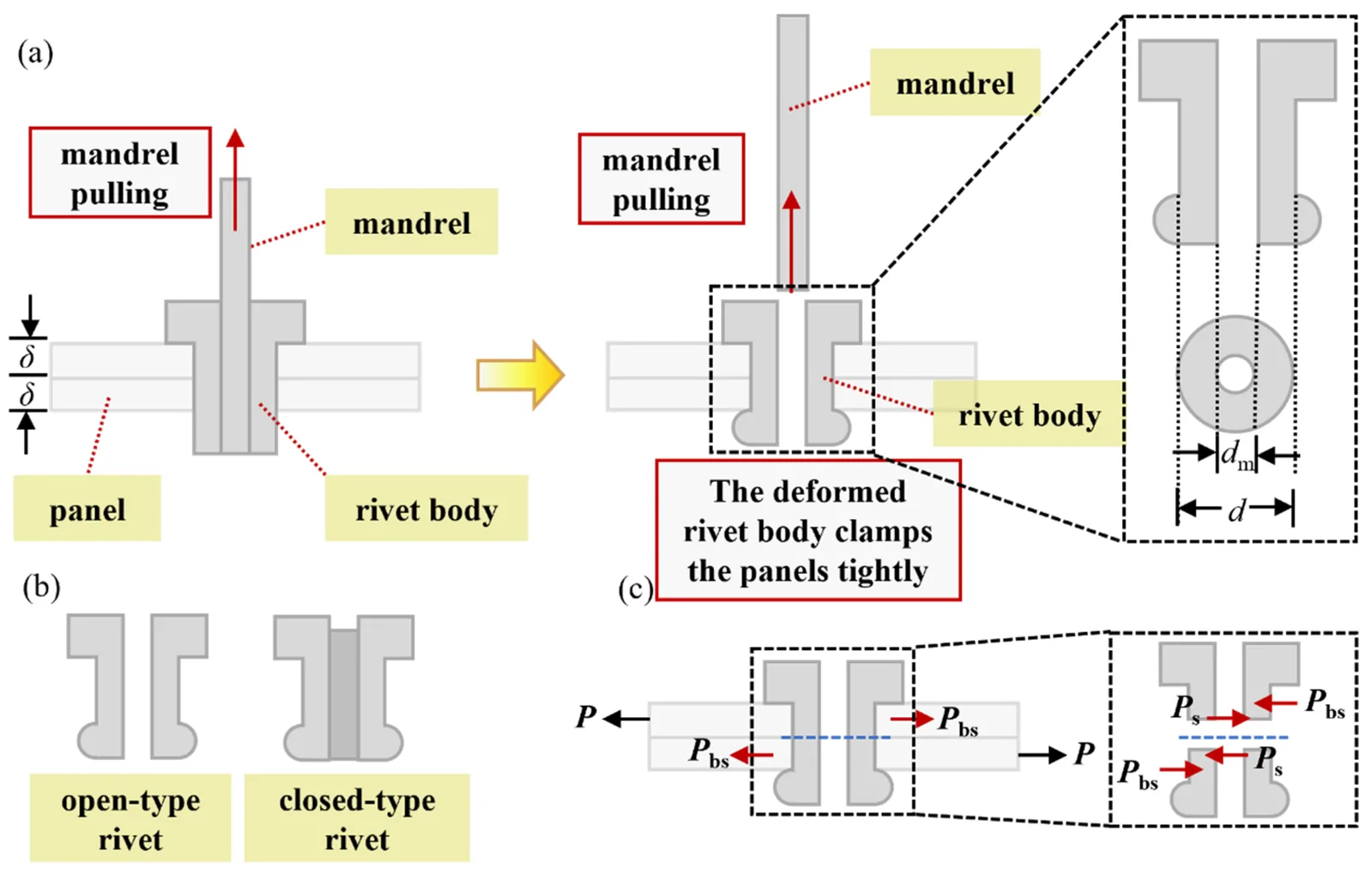
Schematic diagram of blind rivets: (**a**) working schematic of blind rivet; (**b**) types of blind rivets; (**c**) force analysis of blind rivet.

The mechanical properties of the rivet material and the riveted component material shall be close to each other, with matched coefficients of thermal expansion (CTE) as far as possible, and no galvanic corrosion shall occur between the two materials. To ensure that neither the component nor the rivet fails prematurely, the material strength of the rivet shall be equal to that of the component; if the rivet material strength is lower than that of the base plate, the adhesive layer shall be adopted for supplementary reinforcement. For connected components under loading, rivets bear shear force and bearing force. Meanwhile, the mutual extrusion between the rivet shank and the rivet hole subjects the component to bearing stress at the rivet hole position. The force diagram of the blind rivet is shown in Figure 1. If the rivet material strength is lower than the component material strength, the rivet may suffer shear fracture or bearing deformation. If the rivet material strength is higher than the component material strength, the rivet hole may undergo bearing deformation, resulting in rivet loosening and loss of fastening function. For open-type blind rivets, the hollow structure of the rivet shall be taken into account. Based on the matching relationship between the two loads borne by the rivet, the calculation formula for rivet diameter can be derived.

(1) $$\frac{\pi }{4}\left(d^{2}-d_{m}^{2}\right)\tau_{r}=\left(d-d_{m}\right)\delta \sigma_{\mathrm{re}}$$

where *d* is the outer diameter of the rivet body, *d*_m_ is the diameter of the rivet mandrel (i.e., the inner diameter of the rivet body), *δ* is the thickness of the base plate (the patch has the same thickness as the base plate), *σ*_re_ is the bearing strength of the rivet, and *τ*_r_ is the shear strength of the rivet.

Simplification yields:

(2) $$d+d_{m}=\frac{4}{\pi }\frac{\sigma_{\mathrm{re}}}{\tau_{r}}\delta =\frac{4}{\pi }\frac{r_{r}}{k_{r}}\delta$$

where *σ*_re_ = *r*_r_ *σ*_r_, *τ*_r_ = *k*_r_ *σ*_r_, *σ*_r_ is the ultimate strength of the rivet, *r*_r_ is the rivet bearing coefficient, and *k*_r_ is the rivet shear coefficient.

The calculation formula for structural solid rivets is as follows:

(3) $$d=\frac{4}{\pi }\frac{\sigma_{\mathrm{re}}}{\tau_{r}}\delta =\frac{4}{\pi }\frac{r_{r}}{k_{r}}\delta$$

No standard-compliant rivets are applicable for large-thickness riveted structures. The total thickness of a riveted assembly is the sum of the thicknesses of multiple stacked components. As verified by experiments, the following empirical formula is adopted [12], where *∑δ* denotes the total thickness of the clamped assembly (base plate plus patch).

(4) $$d=2\sqrt{\sum \delta }$$

### 2.2. Rivet Distribution

The design of rivet distribution parameters shall account for the influence of rivet hole arrangement on the failure modes of the base plate structure. For a single row of rivets, the distance between the centers of two adjacent rivets is defined as the rivet pitch *t*. The perpendicular distance between the centerlines of two adjacent rivet rows is defined as the row pitch *a*. The perpendicular distance from the centerline of the outermost rivet row to the edge of the component is defined as the edge distance *c*.

**Figure 2:**
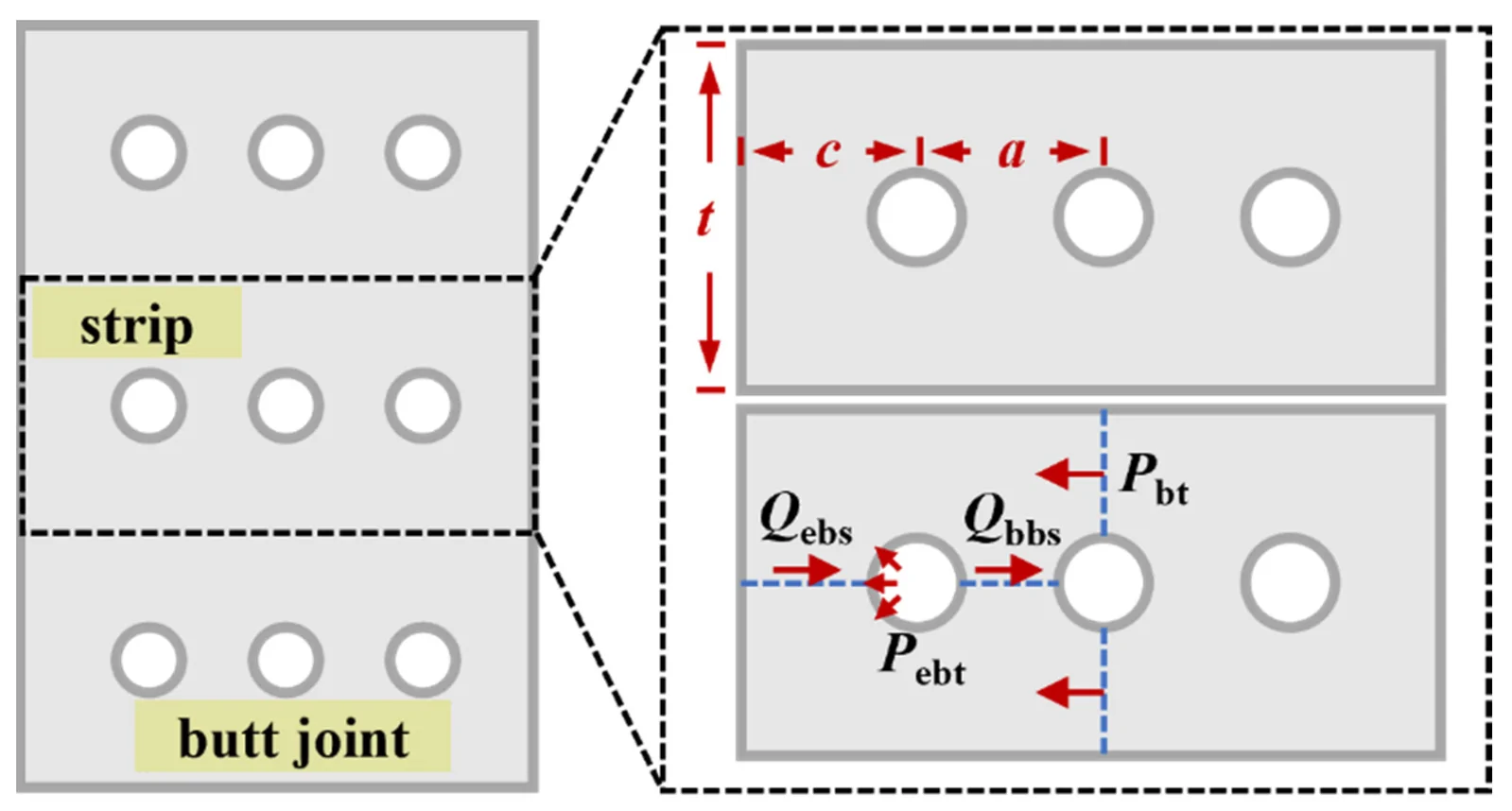
Rivet distribution schematic.

Since all rivet pitches within each row are identical, only a short joint segment with a width equal to one rivet pitch needs to be analyzed. This segment, consisting of a single rivet and the corresponding plate strip of one rivet pitch width, serves as a basic unit of the riveted structure, as shown in Figure 2. When a riveted component is subjected to a tensile load *p*, the plate bears tensile force, the rivet bears shear force, and the plate and rivet are subjected to bearing force from mutual extrusion. The plate also bears shear force at the rivet hole position. Improper rivet arrangement will induce premature failure: an excessively small rivet pitch *t* tends to cause tensile fracture of the plate, while an excessively small edge distance *c* easily leads to shear failure of the plate edge. To prevent independent tensile fracture, shear failure, or bearing failure of the plate, the ultimate tensile load of the plate *P*_bt_, the ultimate shear load of the plate edge *Q*_ebs_, the ultimate shear load of the plate between two adjacent rows *Q*_bbs_, and the ultimate bearing load of the plate *p*_ebt_ shall be equal to each other in rivet layout design.

(1) Calculation of edge distance *c*. For the outermost segment of the base plate, the ultimate bearing load of the plate at a single rivet hole *p*_ebt_ is equal to the ultimate shear load of the plate edge *Q*_ebs_.

(5) $$2\left(c-\frac{d}{2}\right)\delta \tau_{b}=d\delta \sigma_{\mathrm{be}}$$

where *σ*_be_ is the bearing strength of the base plate, and *τ*_b_ is the shear strength of the base plate. Simplification yields:

(6) $$c=\frac{1}{2}\left(1+\frac{\sigma_{\mathrm{be}}}{\tau_{b}}\right)d=\frac{1}{2}\left(1+\frac{r_{b}}{k_{b}}\right)d$$

where *σ*_be_ = *r*_b_ *σ*_b_, *τ*_b_ = *k*_b_ *σ*_b_, *σ*_b_ is the ultimate strength of the base plate, *r*_b_ is the bearing coefficient, and *k*_b_ is the shear coefficient.

(2) Calculation of rivet pitch *t*. For a riveted component with *m* rows of rivets, when the ultimate tensile load of the plate *P*_bl_ is equal to the ultimate bearing load of the plate *p*_ebt_.

(7) $$\left(t-d\right)\delta \sigma_{b}=md\delta \sigma_{\mathrm{be}}$$

for butt joints, *m* denotes the number of rivet rows at the butt interface; for patch-repaired base plates, *m* denotes the number of rivet rows on one side of the perforation. Simplification yields:

(8) $$t=(1+m\frac{\sigma_{\mathrm{be}}}{\sigma_{b}})d=(1+mr_{b})d$$

(3) Calculation of row pitch *a*. For riveted components with more than two rows of rivets, the ultimate shear load of the plate between adjacent rivet rows *Q*_bbs_ is equal to the ultimate shear load of the plate edge *Q*_ebs_.

(9) $$2\left(c-\frac{d}{2}\right)\delta \tau_{b}=2(a-d)\delta \tau_{b}$$

Simplification yields:

(10) $$a=c+\frac{d}{2}=\left(1+\frac{r_{b}}{2k_{b}}\right)d$$

### 2.3. Adhesive Material

For structural repair, blind rivets featuring simple and rapid assembly are adopted to mechanically fasten the patch and the base plate. However, blind rivets exhibit insufficient strength due to their structural form or material properties, so aviation adhesive is employed to compensate for this strength deficiency. To meet aviation safety requirements, fully exploit the strength margin of existing structures, and achieve structural weight reduction, the shear strength of the adhesive layer must match the strength of the base plate and the rivet. The design shall satisfy the condition that the equivalent strength of the hybrid adhesive–rivet structure equals the fracture strength of the plate strip section with one rivet pitch width, i.e.,

(11) $$m\frac{\pi }{4}d^{2}\tau_{\mathrm{req}}+\left(\left(\left(m-1\right)a+2c\right)t-m\pi \frac{d^{2}}{4}\right)\tau_{a}=\sigma_{b}\left(t-d\right)\delta$$

where *τ*_req_ is the equivalent shear strength of the rivet, which covers two components: the shear strength of the hollow rivet body equivalent to a solid rivet, and the equivalent shear strength of the rivet body and rivet mandrel regarded as a homogeneous material.

Substituting Equations (6), (8) and (10) yields:

(12) $$\tau_{a}=\frac{\frac{\delta r_{b}\sigma_{b}}{d}-\frac{\pi }{4}\tau_{\mathrm{req}}}{\left(m+\left(m+1\right)\frac{r_{b}}{2k_{b}}\right)\left(\frac{1}{m}+r_{b}\right)-\frac{\pi }{4}}$$

Satisfaction of this formula ensures mutual strength matching between the hybrid adhesive–rivet structure and the base plate, maximizes the utilization of existing structural strength, and thus achieves structural weight reduction. If the above strength matching requirement cannot be satisfied under existing conditions, the rivet quantity shall be increased to compensate for the strength deficiency.

### 2.4. Rivet Quantity

The strength analysis of hybrid adhesive–rivet structures for patched perforated base plates differs from that of butt joints. The shear strength of the adhesive layer is affected by the bonding area, and the actual matching formula for the shear strength of the adhesive layer is given by:

(13) $$\left[\left(\left(m-1\right)a+c+\frac{D}{2}\right)nt-\frac{1}{2}\cdot \frac{\pi }{4}D^{2}\right]\tau_{a}+N\frac{\pi }{4}d^{2}\left(\tau_{\mathrm{req}}-\tau_{a}\right)=\sigma_{b}\delta n\left(t-d\right)$$

where *n* is the number of rivets in the outermost rivet row, *m* is the number of rivet rows on one half of the patch, and *N* is the total number of rivets on one half of the patch. Generally, *N* < *m n*.

By comparing Equation (11) and Equation (13), it can be observed that the shear strength of the adhesive layer calculated for the base plate repair configuration is higher than that calculated for the butt joint configuration. However, this calculation formula is correlated with the damage diameter *D* of the base plate. For the convenience of strength assessment, the shear strength of the butt joint, i.e., Equation (12), is still adopted, and the rivet quantity is constrained accordingly to ensure post-repair structural safety.

The repair criterion specifies that the patch shall compensate for the load-bearing capacity loss of the base plate caused by perforation. That is, the load capacity loss induced by circular hole cross-section damage equals the total ultimate shear load of rivets on one side of the perforation. Assuming no shear fracture occurs in rivets, and the patch and base plate follow the design principle of equal thickness, identical material and stiffness matching, it is derived that:

(14) $$N\frac{\pi }{4}d^{2}\tau_{\mathrm{raeq}}\geq fD\delta \sigma_{b}$$

where *D* is the diameter of the circular hole, *δ* is the thickness of the base plate, *σ*_b_ is the ultimate strength of the base plate, *N* is the number of rivets on one half of the patch, *d* is the outer diameter of the rivet, *τ*_raeq_ is the equivalent shear strength of the hybrid adhesive–rivet structure, and *f* = 1.5 is the safety factor.

For perforated plates, the shear force borne by the adhesive layer decreases exponentially from the edge of the bonding area to the center. The shear stress of the adhesive layer around the hole is relatively low, so the load-bearing effect of the adhesive layer around the hole is neglected. The calculation formula for the equivalent shear strength of each single rivet is given by:

(15) $$\frac{\pi }{4}d^{2}\tau_{\mathrm{raeq}}=\frac{\left[\left(\left(m-1\right)a+c\right)nt-N\frac{\pi }{4}d^{2}\right]}{N}\tau_{a}+\frac{\pi }{4}d^{2}\tau_{\mathrm{req}}$$

where *n* is the number of rivets in the outermost rivet row, *N* is the total number of rivets on one half of the patch, and *m* is the number of rivet rows on one half of the patch. Note that *N* ≠ *m n*.

(16) $$\tau_{\mathrm{raeq}}=\left[\frac{\left(\left(m-1\right)a+c\right)nt}{N\frac{\pi }{4}d^{2}}-1\right]\tau_{a}+\tau_{\mathrm{req}}$$

Substituting Equations (6), (8), (10), (14) and (16) yields Equation (17), where *N* is rounded up to the nearest integer.

(17) $$\left\{\begin{array}{l} N\geq \left\lceil \frac{T_{0}-N_{0}\tau_{a}}{\tau_{\mathrm{req}}-\tau_{a}}\right\rceil \\ N_{0}=\frac{2n\left(\left(2+\frac{r_{b}}{k_{b}}\right)m-1\right)\left(1+mr_{b}\right)}{\pi }\\ T_{0}=\frac{fD\delta \sigma_{b}}{\frac{\pi }{4}d^{2}} \end{array}\right.$$

## 3. Quasi-Static Tensile Failure Tests and Numerical Simulation

### 3.1. Quasi-Static Tensile Failure Tests

#### 3.1.1. Specimen Design

Two types of rivets and two types of adhesives are selected to verify strength matching. The first rivet is Grade 11 open-type blind rivet specified in GB 12618 [13]. After riveting, the rivet body remains hollow and is made of 5056 aluminum alloy, with an equivalent shear strength of 100 MPa when converted into an equivalent solid rivet. The second rivet is the Cherry CR3212 aerospace structural blind rivet. After installation, the rivet body and mandrel form a solid assembly. According to the manufacturer’s specification, its shear strength is 345 MPa; the rivet body is manufactured from 5056 aluminum alloy and the mandrel from 8740 alloy steel [8]. Two adhesives are adopted for the adhesive layer. One is Lord 320/322 two-part epoxy adhesive with a mixing ratio of 1:1. The recommended adhesive layer thickness ranges from 0.50 mm to 0.76 mm, and 0.50 mm is adopted in this work, with a shear strength of 11.7 MPa [14]. The other adhesive is Araldite-2015 two-part epoxy adhesive at a mixing ratio of 1:1. Its suggested bondline thickness is 0.05–0.10 mm, and 0.10 mm is selected here, with a shear strength of 17.9 MPa [15]. Among the selected blind rivets, the Cherry aerospace blind rivet is defined as the high-strength rivet, while the GB 12618.1 blind rivet is the low-strength rivet. For the joint specimens, Araldite-2015 and Lord 320/322 are used; Araldite-2015 serves as the high-strength adhesive and Lord 320/322 as the low-strength adhesive. Joint specimens combining high-strength rivet with low-strength adhesive and low-strength rivet with high-strength adhesive are designed to investigate the load-bearing capacity under different adhesive–rivet matching configurations.

**Figure 3:**
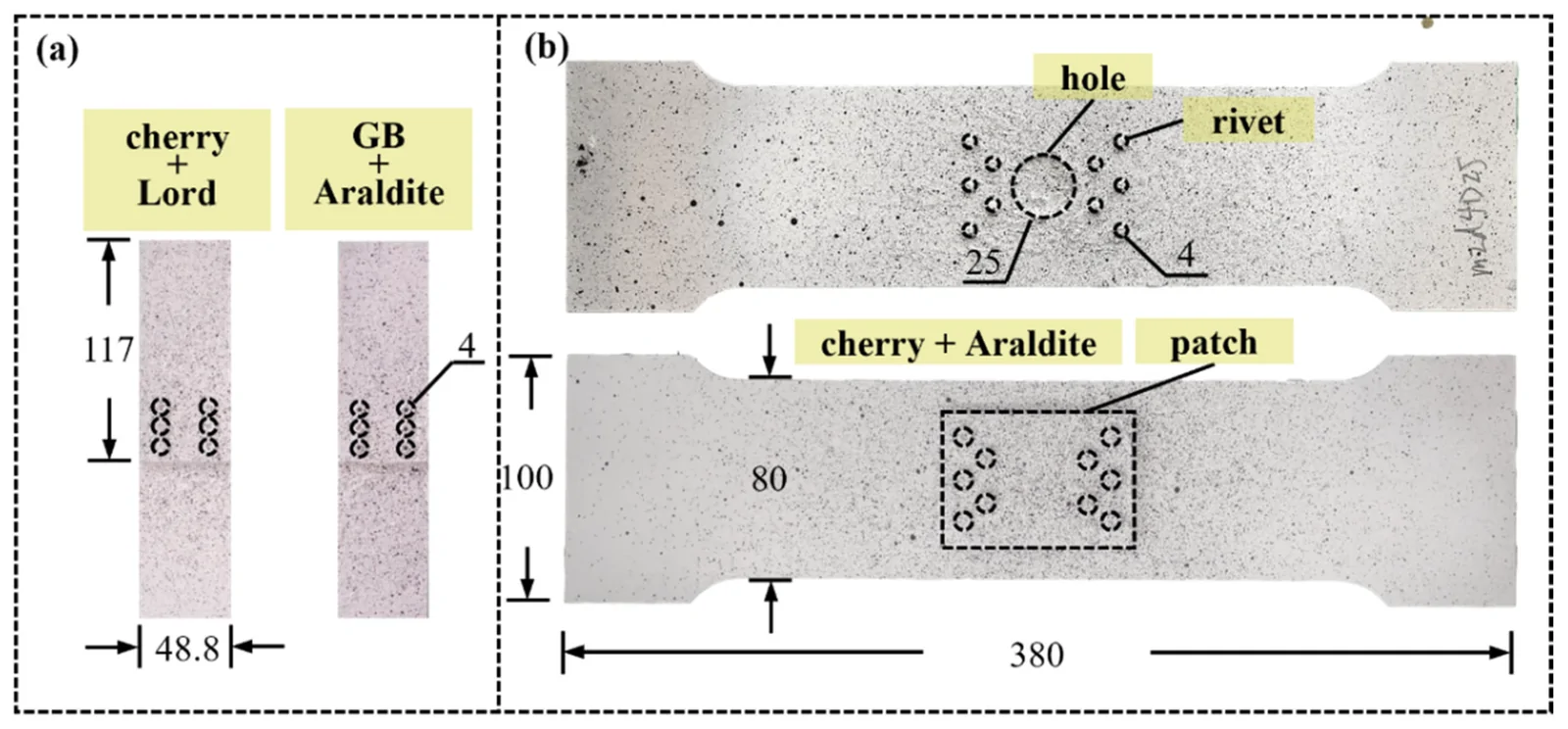
Specimen design: (**a**) butt joint specimen; (**b**) hybrid adhesive–rivet repaired specimen.

For 5xxx-series aluminum alloy, the bearing coefficient *r*_r_ = 1.6 [16] and the shear coefficient *k*_r_ = 0.64. For 2xxx-series aluminum alloy, the bearing coefficient *r*_b_ = 1.7 [17] and the shear coefficient *k*_b_ = 0.634. Substituting these parameters into the formulas in Section 2.2, butt joint specimens with *m* = 2, 3, and rivet diameters *d* = 4.0 mm and 4.8 mm are designed, as listed in Table 1 and Figure 3a.

The butt joints are fabricated from 2A12-T4 aluminum alloy, which is widely used for aircraft panels. The geometric dimensions are dependent on rivet diameter, rivet pitch, edge distance and row pitch. The size of each single plate ranges from 95 mm × 35 mm to 140 mm × 62 mm, and all plates have a uniform thickness of 2 mm. Two plates are joined at the rivet holes to form a butt joint. Spacers with a length of 50 mm and thickness of 2 mm are mounted on both sides to restrain significant eccentric bending moment under tension, which would otherwise compromise the test results.

As described in Section 2.4, the patched perforated base plate specimens adopt high-strength Cherry CR3212 blind rivets and Araldite-2015 adhesive layer, so as to prevent structural failure caused by shear fracture of rivets or adhesive layer after adhesive–rivet hybrid repair.

The staggered rivet arrangement, which is commonly used in riveted repair, is adopted. For one-sided rivet layout, *m* = 2, where *N* = 5 and *m* = 3 where *N* = 7. By inverse calculation using Equation (17), the maximum allowable circular hole diameters corresponding to different rivet diameters *d* are 25 mm, 36 mm and 43 mm, respectively. The objective is to verify whether the hybrid adhesive–rivet repair meets strength requirements under these hole diameters. The test matrix is summarized in Table 2 and Figure 3b. Flat specimens with corresponding hole diameters and intact flat specimens are fabricated for comparison to evaluate the repair performance.

The base plate of repaired specimens adopts a dog-bone configuration. The overall dimension of the base plate is 380 mm × 100 mm, the width of the central test section is 80 mm, and each gripping section on both sides has a length of 50 mm, connected via fillets with a radius of 25 mm. The patch dimensions depend on rivet diameter, rivet pitch, edge distance, row pitch and circular hole diameter. The patch sizes range from 77 mm × 52.8 mm to 103 mm × 73.2 mm Both the base plate and patches have a thickness of 2 mm and are manufactured from 2A12-T4 aluminum alloy.

#### 3.1.2. Test Setup

**Figure 4:**
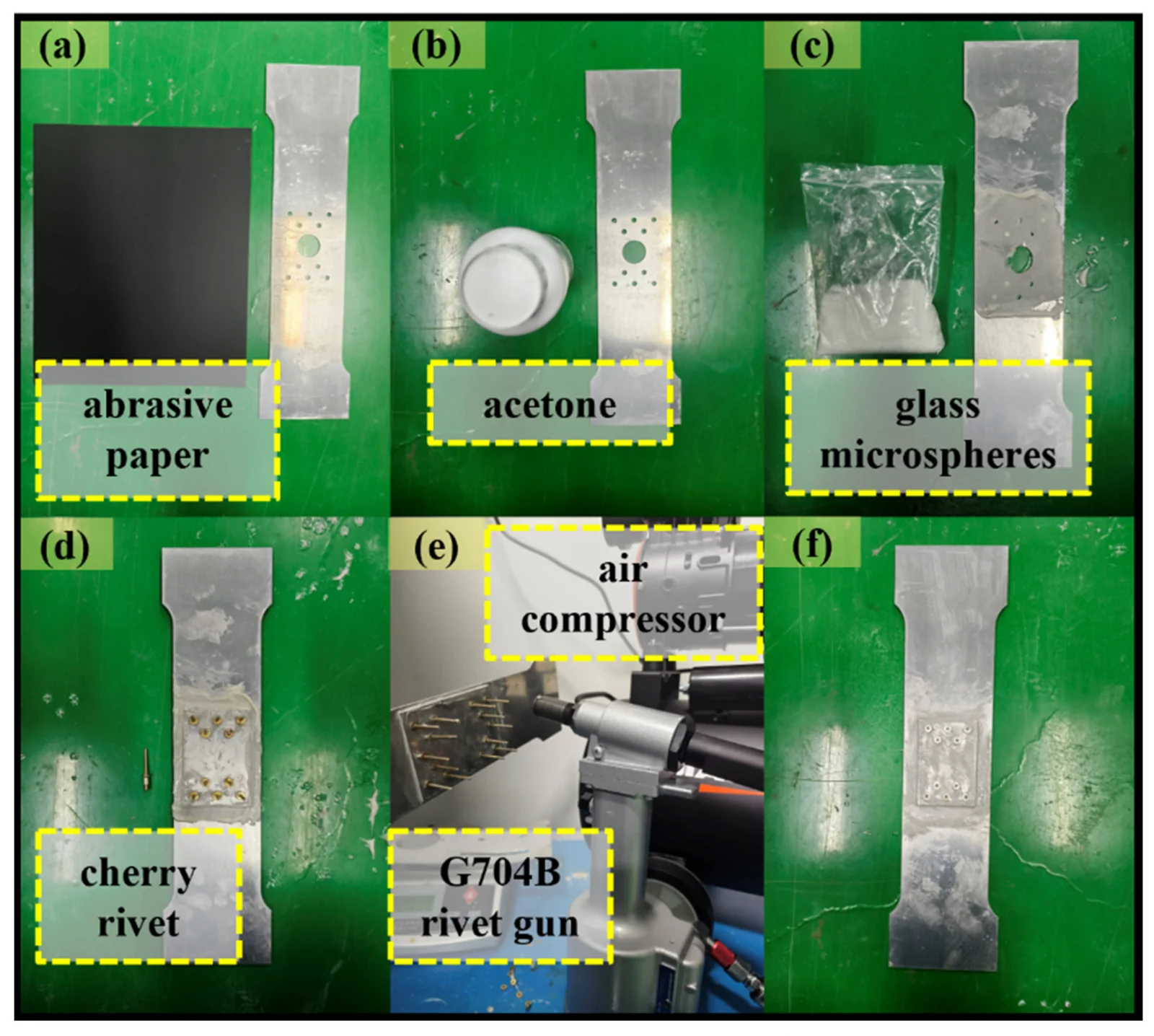
Specimen fabrication process: (**a**) Sand-paper polishing; (**b**) Acetone cleaning; (**c**) Glass-microsphere spraying and adhesive coating; (**d**) Rivet installation; (**e**) Rivet-gun riveting; (**f**) Specimen preparation completed.

Specimen fabrication consists of four procedures: surface grinding, cleaning, adhesive bonding and riveting, as illustrated in Figure 4. The grinding process removes the oxide layer on the aluminum alloy surface and increases surface roughness. In the cleaning procedure, acetone solution is adopted to eliminate surface grease and grinding debris. After wiping, the specimens are placed to allow natural volatilization of acetone. For adhesive bonding, the designated adhesive is evenly applied onto the aluminum alloy surface with a glue gun. Tiny glass beads are sparsely distributed over the adhesive layer, followed by compaction using the patch, and excess squeezed adhesive fillet is wiped off. A 400 mL two-component glue gun is used for Araldite-2015, while a 50 mL two-component glue gun is adopted for Lord 320/322. In the riveting procedure, rivets are installed onto the bonded specimens and set with the corresponding riveting tools. Subsequently, the specimens are cured integrally for 24 h. The Grade 11 rivets conforming to GB standard are installed using the Anzi SSC150 double-hand rivet gun, and Cherry rivets are fitted with the G704B rivet gun equipped with an H701B-456 pulling head.

**Figure 5:**
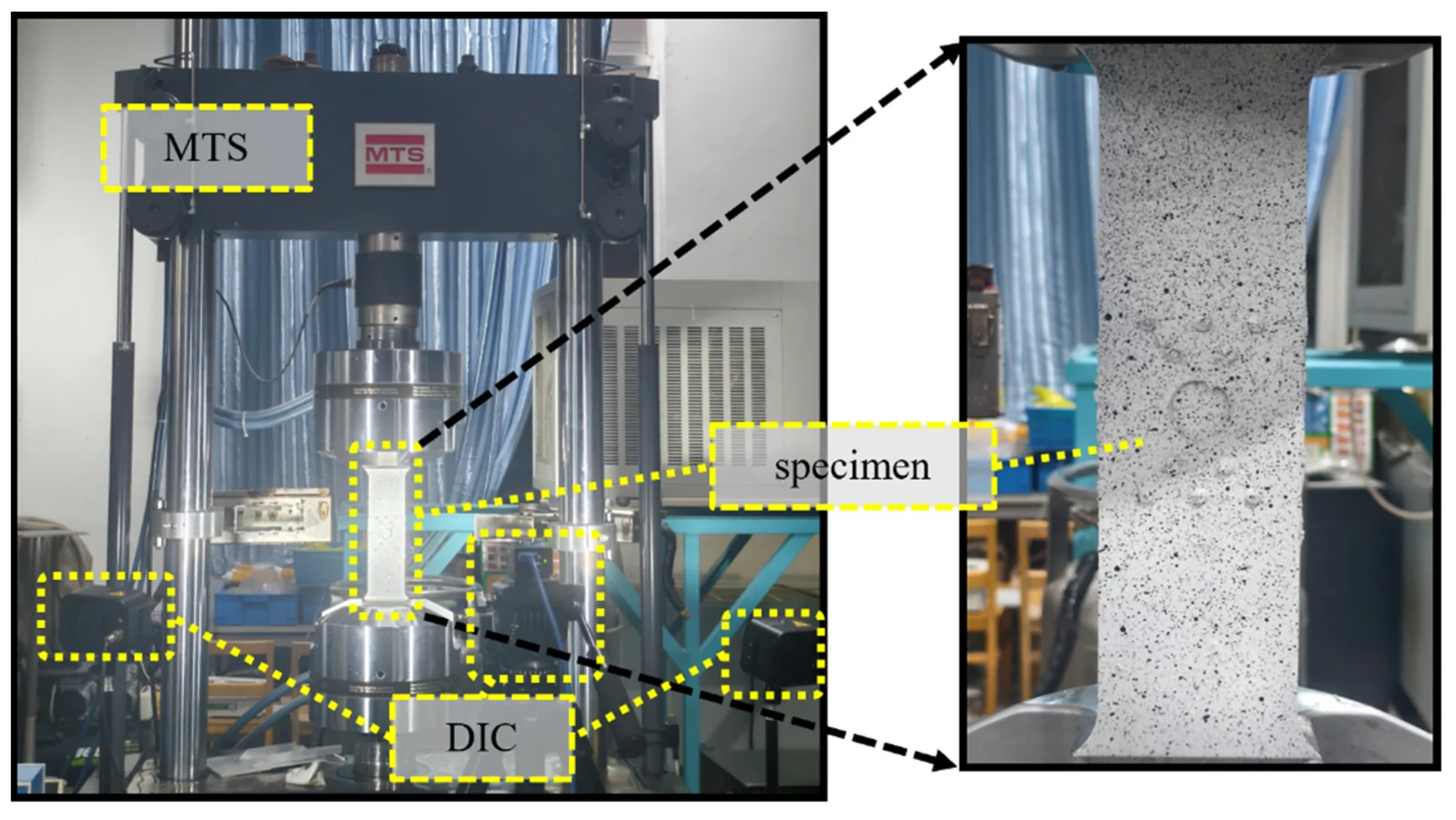
Test setup schematic.

The test setup comprises an MTS hydraulic fatigue testing machine, which applies quasi-static tensile loading at a displacement rate of 1 mm/min. The specimen joint section is clamped by the upper and lower grips of the testing machine to provide uniform tensile stress. An industrial control computer records the load–displacement curves throughout the tensile test. A digital image correlation (DIC) system captures the structural failure evolution of specimens with an image acquisition interval of 500 ms. Auxiliary lights on both sides are adjusted to optimize illumination. The specimen surface is uniformly coated with white primer and speckled with black random speckles. For patched repair specimens, speckles are applied on the side without the patch, enabling the DIC software Vic-2D 7.2.0 to accurately analyze and compute the evolution of surface strain contours. The test scene is shown in Figure 5. For each configuration, three specimens were tested (n = 3).

### 3.2. Numerical Simulation

#### 3.2.1. Material Constitutive

The ductile damage criterion [18,19] serves as a phenomenological model describing ductile fracture in metals, effectively simulating the nucleation, growth, and coalescence of voids during tensile deformation. This criterion consists of two distinct phases: ductile damage initiation and ductile damage evolution.

The ductile damage initiation characterizes the onset of material damage, which is typically governed by three key variables: equivalent plastic strain, stress triaxiality, and strain rate.

(18) $${\bar{\varepsilon }}_{D}^{pl}\left(\eta ,{\dot{\bar{\varepsilon }}}^{pl}\right)$$

The stress triaxiality (*η*) is defined as η=−p/q, where *p* is the pressure stress, *q* is the Mises equivalent stress and ${\bar{\varepsilon }}^{pl}$ is the equivalent plastic strain rates.

State variables are defined to characterize the damage initiation criterion. Ductile damage in metallic materials is considered to initiate when $\omega_{D}=1$ is satisfied.

(19) $$\omega_{D}=\int \frac{d{\bar{\varepsilon }}^{pl}}{{\bar{\varepsilon }}_{D}^{pl}\left(\eta ,{\dot{\bar{\varepsilon }}}^{pl}\right)}=1$$

In finite element analysis, the computational method for each analysis step increment is as follows:

(20) $$\Delta \omega_{D}=\frac{\Delta {\bar{\varepsilon }}^{pl}}{{\bar{\varepsilon }}_{D}^{pl}\left(\eta ,{\dot{\bar{\varepsilon }}}^{pl}\right)}\geq 0$$

The ductile damage evolution characterizes the progressive stiffness degradation of metallic materials, which can be defined as either linear or exponential. The degradation rate can be quantified using either fracture energy or displacement at failure.

The overall damage variable *D* quantifies the cumulative damage in the structural element, where *D* = 1 indicates element failure and removal. The failure process is characterized by both fracture energy and critical displacement, with their relationship established in [20].

(21) $$G_{f}=\int_{{\bar{\varepsilon }}_{oi}}^{{\bar{\varepsilon }}_{f}^{pl}}L\sigma_{y}d{\bar{\varepsilon }}^{pl}$$

The cohesive zone model (CZM) [7] is adopted to simulate the initiation and propagation of internal cracks within the adhesive layer. A cohesive damage model following the bilinear traction-separation law is employed to characterize the damage initiation and evolution of the adhesive layer. The damage variable *D* is used to describe the damage state of the adhesive material. The damage variable *D*, ranging from the onset of adhesive damage to final failure, is defined as:

(22) $$D=\frac{\delta^{f}\left(\delta -\delta^{0}\right)}{\delta^{0}\left(\delta^{f}-\delta^{0}\right)}$$

where *δ*^0^ is the displacement at damage initiation, *δ* is an arbitrary displacement during damage evolution, and *δ*^f^ is the displacement at complete failure. The cohesive elements are fully failed when *D* = 1.

After introducing the damage state variable, the expression for interfacial traction is written as:

(23) $$\begin{array}{l} t_{n}=\left\{\begin{array}{cc} \left(1-D\right)t_{n}^{0}, & t_{n}^{0}\geq 0\\ t_{n}^{0}, & t_{n}^{0}<0 \end{array}\right.\\ t_{s}=\left(1-D\right)t_{s}^{0}\\ t_{t}=\left(1-D\right)t_{t}^{0} \end{array}$$

where *t*_n_^0^, *t*_s_^0^ and *t_t_*^0^ denote the interfacial tractions of the adhesive layer prior to damage initiation.

Compared with the maximum nominal stress criterion, the quadratic nominal stress criterion explicitly accounts for interactions among the normalized traction components, making it more suitable for the mixed-mode stress state involving coupled shear and peel stresses considered in this study. The expression for damage initiation based on the quadratic stress criterion is given by:

(24) $$\begin{array}{cc} {\left(\frac{t_{n}}{t_{n}^{0}}\right)}^{2}+{\left(\frac{t_{s}}{t_{s}^{0}}\right)}^{2}+{\left(\frac{t_{t}}{t_{t}^{0}}\right)}^{2}=1, & t_{n}=\left\{\begin{array}{cc} \left(1-D\right)t_{n}^{0}, & t_{n}^{0}\geq 0\\ t_{n}^{0}, & t_{n}^{0}<0 \end{array}\right. \end{array}$$

The Benzeggagh–Kenane (B–K) criterion is adopted for damage evolution, the formula for which is written as:

(25) $$G_{I}^{C}+\left(G_{\mathrm{II}}^{C}-G_{I}^{C}\right){\left(\frac{G_{\mathrm{II}}^{C}+G_{\mathrm{III}}^{C}}{G_{I}^{C}+G_{\mathrm{II}}^{C}+G_{\mathrm{III}}^{C}}\right)}^{\eta }=G^{C}$$

where *G*_i_^C^ (*i* = I, II, III) is the critical strain energy release rate, G^C^ is the total critical strain energy release rate, and *η* is the interaction parameter with a value of 1.5 [21].

The material properties of 2A12-T4 aluminum alloy for base plates and patches, as well as Cherry CR3212 aerospace blind rivets consisting of 5056 aluminum alloy and 8740 alloy steel, are summarized in Table 3. The material parameters of the two-part epoxy adhesive Araldite-2015 are listed in Table 4.

#### 3.2.2. Model Setups

**Figure 6:**
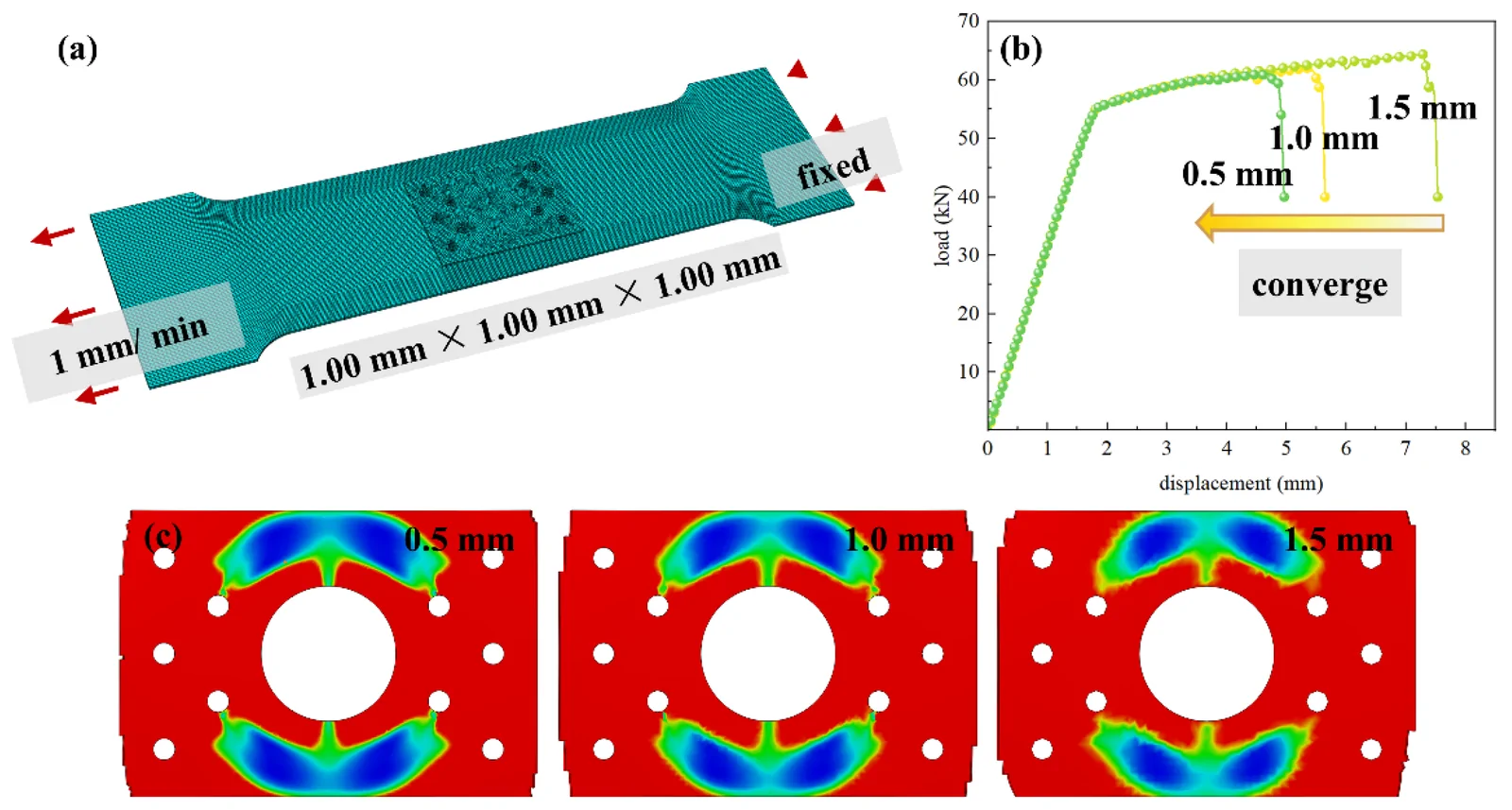
Numerical simulation model: (**a**) finite element model; (**b**) mesh independence validation; (**c**) damage initiation location.

Based on the commercial finite element software Abaqus, a numerical model of hybrid adhesive–rivet repaired structure is established. The overall dimension of the model is 380 mm × 100 mm × 2 mm. The mesh size of the central hybrid adhesive–rivet joint region is 1.0 mm × 1.0 mm × 1.0 mm, while the mesh size for the two lateral regions is 2.0 mm × 2.0 mm × 1.0 mm. A solid adhesive layer with a thickness of 0.1 mm is constructed between the base plate and the patch, which is assigned cohesive element properties and cohesive material properties. The adhesive layer is connected with plates via shared nodes. Solid rivets are assembled into the rivet holes of the patch and base plate. General contact is defined between rivets and rivet holes; the friction coefficient for tangential behavior is set to 0.2 [22], and hard contact is adopted for normal interaction. Reference points are created on both ends of the base plate. One end is fully fixed with displacement constraints, and the displacement loading rate is controlled at 1 mm/min. The finite element model is shown in Figure 6a. In the riveting simulation, no initial rivet preload, installation-induced interference, residual stress, or clamp-up was explicitly modeled. This simplification was adopted because the present study focuses on the ultimate tensile residual strength of the repaired structure under quasi-static loading, for which the rivets mainly sustain shear and bearing loads near failure. The rivet shank–hole wall contact was included to transmit contact and bearing forces during loading, but it does not reproduce the full mechanical state produced by blind-rivet installation. Therefore, installation-induced residual stresses, local interference, and clamp-up effects are neglected in the present model.

A sensitivity analysis was also conducted to evaluate the effect of the friction coefficient between the rivet and the rivet hole. Taking a friction coefficient of 0.20 as the baseline, variations of ±20% were considered for the m2d4D25 configuration. The predicted failure loads corresponding to friction coefficients of 0.20, 0.16, and 0.24 were 62.02, 61.86, and 62.12 kN, respectively. Compared with the baseline value of 62.02 kN, the deviations were only −0.26% and 0.16%, respectively, indicating that the predicted failure load is highly insensitive to reasonable variations in the rivet–hole friction coefficient.

#### 3.2.3. Model Validation

(1) Mesh Independence Validation

On the basis of the mesh size of 1.0 mm × 1.0 mm × 1.0 mm, two additional mesh sizes of 0.5 mm × 0.5 mm × 1.0 mm and 1.5 mm × 1.5 mm × 1.0 mm are adopted for the central hybrid adhesive–rivet joint region. The mesh size of 2.0 mm × 2.0 mm × 1.0 mm is maintained for the remaining regions on both sides. The corresponding load–displacement curves of finite element models are presented in Figure 6b. The load–displacement curves exhibit obvious convergence tendency when the mesh size decreases from 1.5 mm to 0.5 mm. Discrepancies exist in displacement, while the failure loads are nearly consistent. The errors of failure load for the 1.0 mm mesh model relative to the 1.5 mm and 0.5 mm mesh models are 4.20% and −1.05%, respectively. Furthermore, the computational efficiency of the 1.0 mm mesh is superior to that of the 0.5 mm mesh. Consequently, the mesh size of 1.0 mm × 1.0 mm × 1.0 mm is selected for subsequent simulations.

To further verify the mesh convergence of the cohesive zone model, the maximum adhesive shear stress, peel traction, damage initiation location, equivalent plastic strain (PEEQ) around the rivet holes at the onset of adhesive damage, and the residual bonded-area ratio (defined as the ratio of the undebonded adhesive area to the total adhesive area) obtained using three different mesh sizes are compared in Table 5. In addition, the maximum, minimum, and total rivet loads before adhesive failure are summarized in Table 6.

To evaluate the sensitivity of local responses to mesh size, the maximum adhesive shear stress, maximum peel traction, damage initiation location, PEEQ near the rivet holes, residual bonded-area ratio, and rivet load distribution were compared using mesh sizes of 0.5, 1.0, and 1.5 mm. The results show that the differences between the 1.0 mm and 0.5 mm meshes in the maximum shear stress, maximum peel traction, PEEQ, and residual bonded-area ratio are only 0.06%, 1.58%, 1.85%, and 0.54%, respectively, indicating that the main local responses are essentially converged. In contrast, the 1.5 mm mesh exhibits more pronounced deviations in the local plastic response around the rivet holes and in the rivet load distribution. Specifically, the maximum individual rivet load and the total rivet load are 38.85% and 64.86% higher, respectively, than those obtained with the 0.5 mm mesh. For all three mesh sizes, adhesive damage initiates at the outer edges of the adhesive layer and subsequently propagates inward, indicating that variations in mesh size do not alter the primary failure mechanism, as shown in Figure 6c.

Overall, both the global and local response indicators obtained with the 1.0 mm mesh show good convergence and are close to those obtained with the finer 0.5 mm mesh. Therefore, the 1.0 mm mesh provides sufficient numerical accuracy for the strength assessment conducted in this study while maintaining reasonable computational efficiency, and was thus selected for the subsequent analyses.

(2) Material sensitivity analysis

A material sensitivity analysis was conducted for the cohesive zone model by varying *t*_n_^0^, *t*_s_^0^, *G*_I_^C^, *G*_II_^C^ and *η* by ±20% relative to their baseline values listed in Table 4. For the *m*2*d*4*D*25 configuration, the corresponding adhesive damage initiation load, maximum failure load, failure displacement, and residual bonded-area ratio—defined as the ratio of the undebonded adhesive area to the total adhesive area—were calculated. The results are summarized in Table 7.

To further quantify the sensitivity, a normalized sensitivity coefficient, *S*_x_, is defined as follows:

(26) $$S_{X}=\frac{Y_{1.2}-Y_{0.8}}{0.4Y_{0}}$$

where *Y*_1.2_ is the numerical result obtained using 1.2 times the baseline parameter value, *Y*_0.8_ is the numerical result obtained using 0.8 times the baseline parameter value, and *Y*_0_ is the numerical result corresponding to the baseline parameter value. The calculated normalized sensitivity coefficients are presented in Table 8.

The CZM parameter sensitivity analysis indicates that the overall ultimate load-carrying capacity of the m2d4D25 configuration is insensitive to parameter perturbations. When *t*_n_^0^, *G*_I_^C^, *G*_II_^C^, and the B–K parameter *η* are varied by ±20%, the maximum failure load changes by only −0.88% to +0.72%. Moreover, the normalized sensitivity coefficients corresponding to the maximum failure load, ∣*SP*_u_∣, are all below 0.05, demonstrating that the model exhibits good robustness in predicting the overall failure load.

By contrast, the local damage evolution of the adhesive layer is more sensitive to several CZM parameters. Among them, *G*_II_^C^ exhibits the highest overall sensitivity, with normalized sensitivity coefficients of 0.126, 0.242, and 0.345 for the damage initiation load, failure displacement, and damaged-area ratio, respectively. The sensitivity of *t*_s_^0^ to the damaged-area ratio reaches 0.182, indicating that the adhesive shear strength and Mode-II fracture energy are the primary parameters governing the propagation of adhesive damage. The B–K parameter and mainly affect the local damage extent; whereas, *G*_I_^C^ has the weakest overall influence.

Overall, the sensitivity of the local damage response can be ranked as *G*_II_^C^ > *t*_s_^0^ > *η* > *t*_n_^0^ > *G*_I_^C^. This indicates that the adhesive–rivet hybrid repaired structure exhibits a distinctly shear-dominated damage behavior, with local adhesive damage primarily controlled by *G*_II_^C^ and *t*_s_^0^. In contrast, the global load-carrying capacity remains stable within a reasonable range of CZM parameter perturbations, demonstrating that the model provides reliable predictions of the overall failure load.

(3) Experiment and simulation comparison

**Figure 7:**
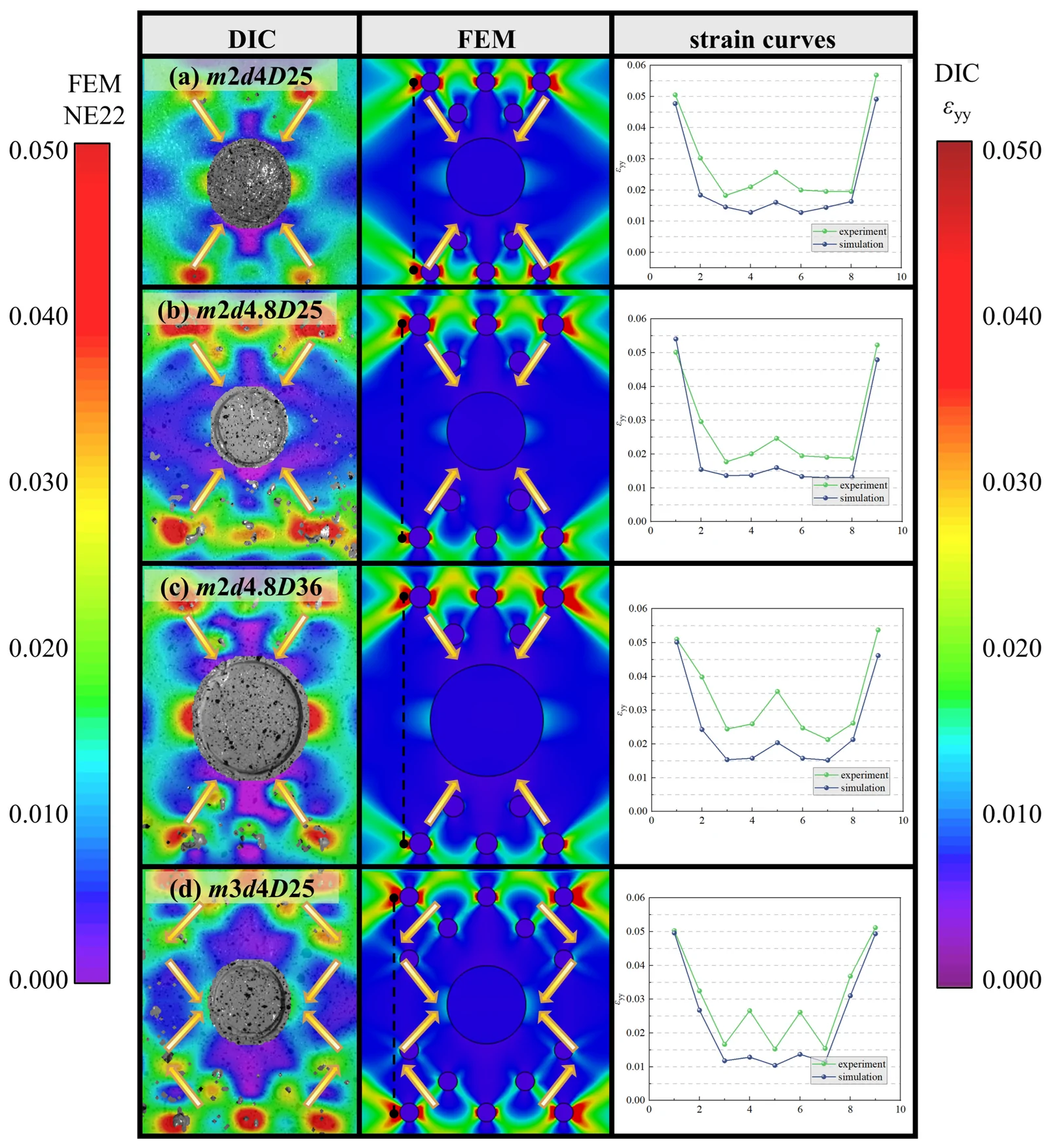
Comparison of strain contours between experiments and numerical simulations.

The strain-field distributions obtained from the simulations and experiments are compared in Figure 7. Representative configurations were selected for comparison, and the experimental and numerical strain contours exhibit similar overall distributions and plastic deformation patterns. In the simulations, the nominal strain in the *y*-direction, *NE*22, was extracted; whereas, the experiments provided the Green–Lagrange strain in the *y*-direction, *ε*_yy_, measured by DIC. These two strain measures are not strictly identical; however, their difference is negligible within the small-strain range considered here (*NE*22 ≤ 5%). Therefore, a direct comparison is considered reasonable.

The numerical results exhibit a more localized high-strain region with a smaller spatial extent than that observed experimentally. In particular, higher strain concentrations are predicted around the rivet holes, which can be attributed to the discretized finite element representation and the resulting localization of deformation near geometric discontinuities. To further quantify the agreement, strain values around the rivet holes were extracted from both the experimental and numerical results. Nine sampling points were selected between the two outermost rivets in the outermost rivet row, and the corresponding-direction strains were compared. The numerical and experimental results show good agreement on both sides of the rivet holes, although the numerical strain is lower than the experimental value in the central region. Overall, both results exhibit consistent strain variation trends, supporting the capability of the finite element model to reproduce the strain distribution and deformation characteristics of the repaired structure.

(27) $$\varepsilon_{\mathrm{yy}}^{GL}=NE22+\frac{1}{2}{\left(NE22\right)}^{2}\approx NE22,\;NE22\leq 5\%$$

**Figure 8:**
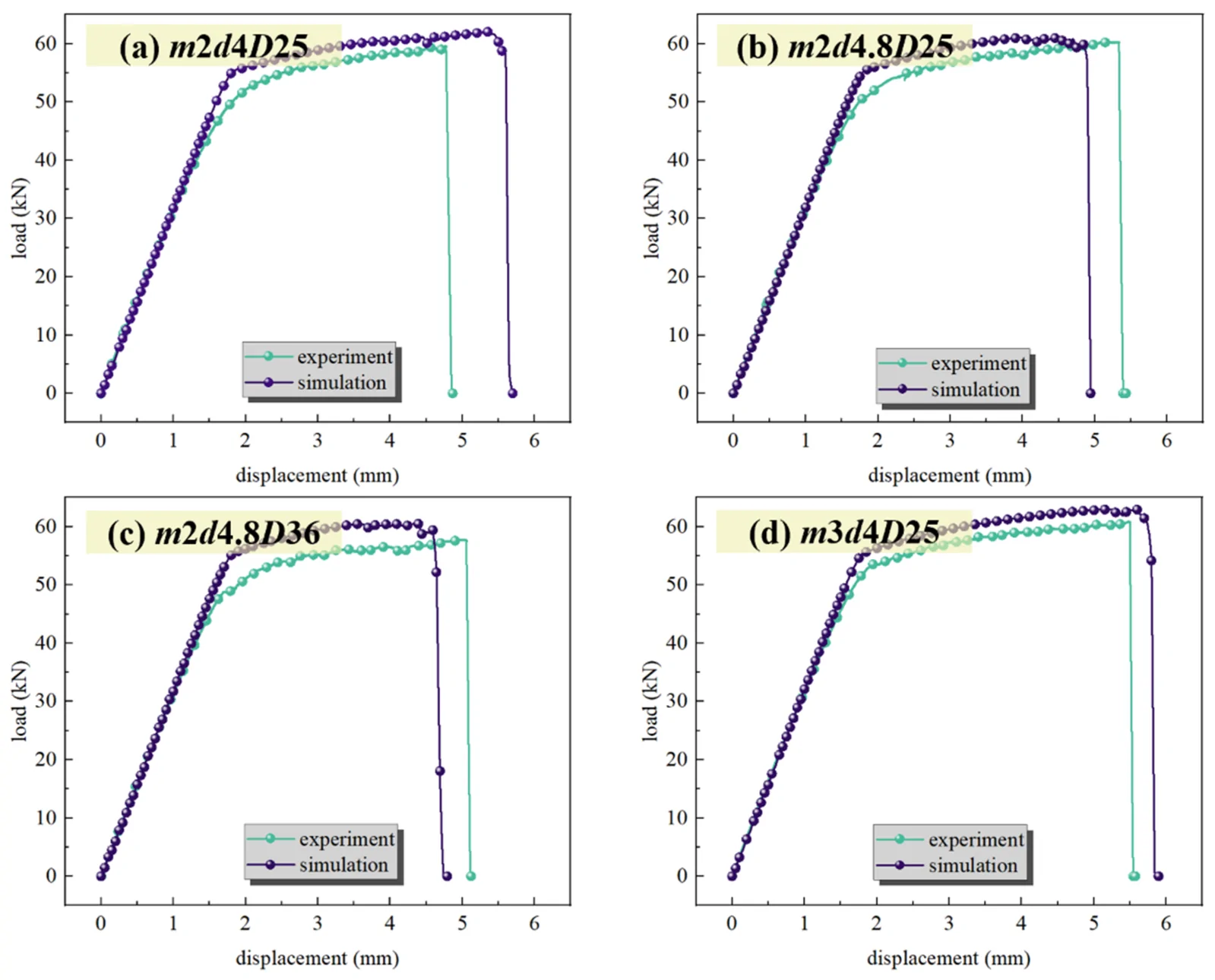
Comparison of load–displacement curves between experiments and numerical simulations.

**Figure 9:**
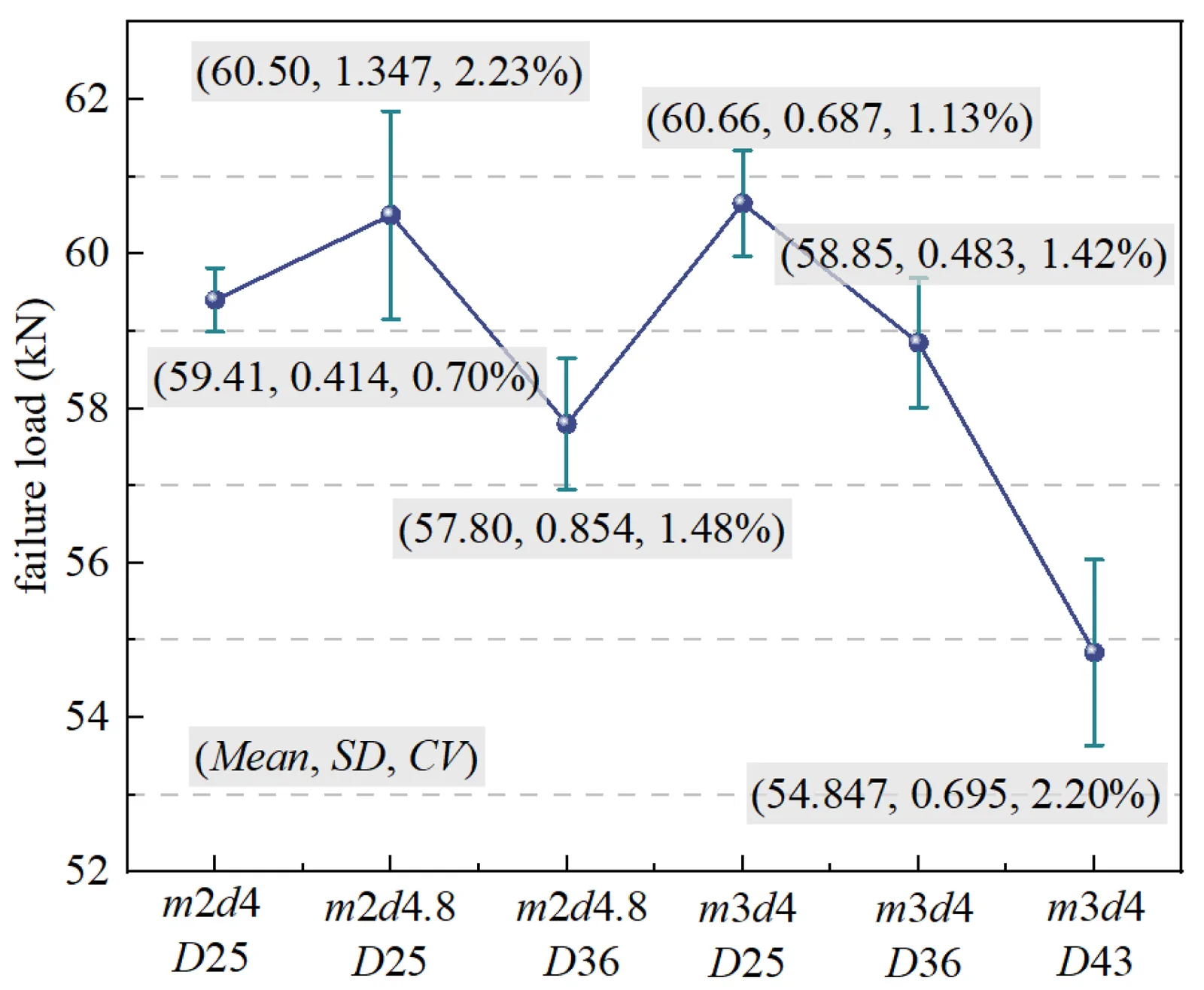
Experimental results with error bars.

The load–displacement curves obtained from numerical simulations and experiments are compared in Figure 8. For selected working conditions, the simulated and experimental load–displacement curves share similar trends. At the initial stage, the curve rises linearly within the elastic regime. After entering the strain hardening stage, the growth rate of the curve gradually slows down, and the load drops sharply once structural failure occurs. Overall, the discrepancy in failure load between simulation and experiment is minor, with a maximum error of 5.02%, which satisfies the accuracy requirement. A representative load–displacement curve was selected from the experimental results, while the overall test results with the corresponding error bars are presented in Figure 9. The values in parentheses represent the mean value, sample standard deviation, and coefficient of variation, respectively.

## 4. Mechanical Characteristics and Failure Analysis of Structures Repaired by Hybrid Adhesive–Rivet Joints

### 4.1. Load-Bearing Characteristics Under Hybrid Adhesive–Rivet Interaction

In tensile failure tests of joint specimens, the failure modes under various working conditions are dominated by the failure of the adhesive layer and rivets. Under non-collinear tensile load, bending moment is generated in butt joints, leading to bending deformation at the joint region. The adhesive layer near the joint edge firstly undergoes debonding failure. Afterwards, the rivets and residual central adhesive layer sustain shear force and bending moment until complete failure of both rivets and adhesive layer.

**Figure 10:**
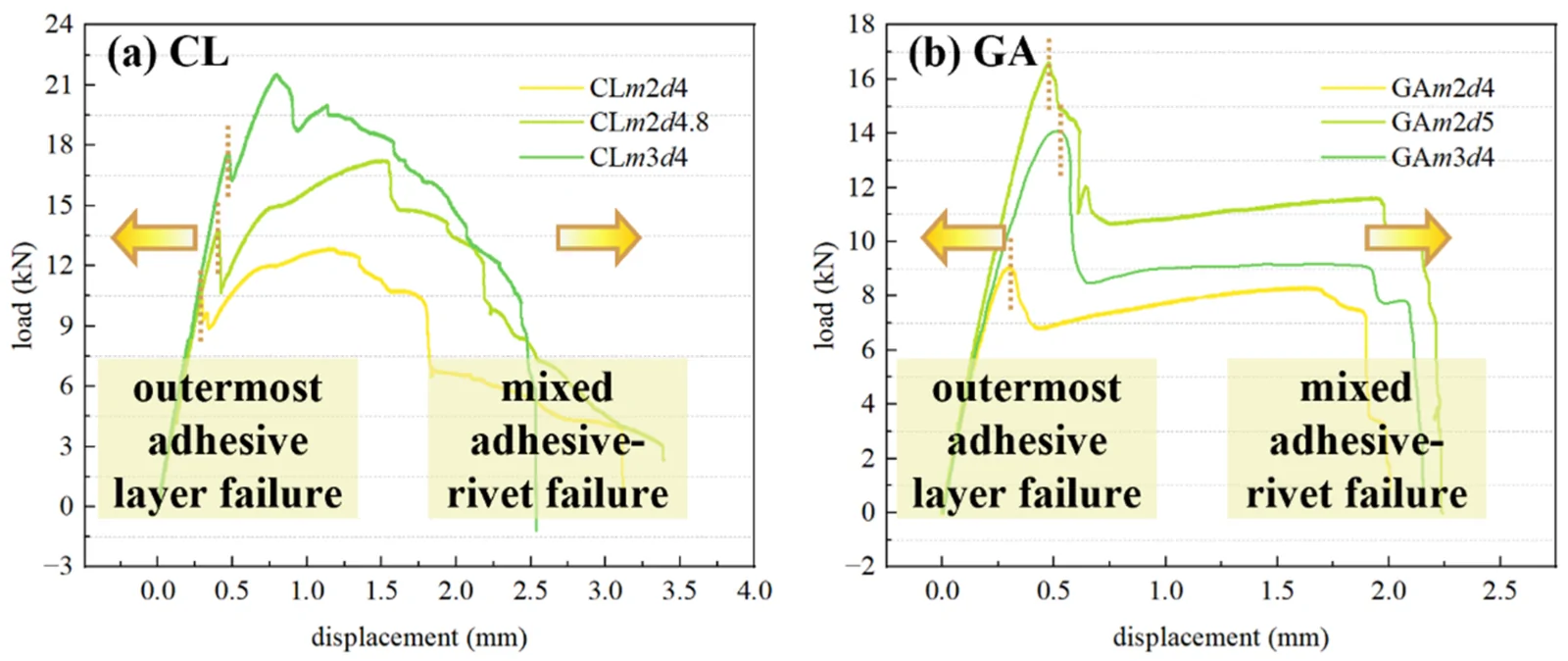
Load–displacement curves of butt joint specimens: (**a**) specimens with Cherry rivets and Lord adhesive; (**b**) specimens with GB rivets and Araldite adhesive. Arrows denote the division of failure processes for the bonded-riveted structure. The left direction represents the outermost adhesive layer failure, and the right direction denotes the mixed adhesive-rivet failure.

The load–displacement curves of joint specimens with different adhesive–rivet matching configurations exhibit double peak loads, which correspond to the failure process described above. The edge adhesive layer debonds first, resulting in an instantaneous load drop of the butt joint. Subsequently, the rivets and residual adhesive layer continue to bear the load, and the load rises gradually until shear fracture of rivets occurs, whereby the butt joint loses its load-bearing capacity. Accordingly, the first load drop is governed by the adhesive strength, while the second load drop mainly depends on the rivet strength. Distinct load–displacement responses are observed for combinations of high-strength rivet with low-strength adhesive and low-strength rivet with high-strength adhesive. Figure 10a presents the configuration of high-strength Cherry rivet matched with low-strength Lord adhesive. The first peak load is relatively low owing to the low strength of Lord adhesive, and the second peak load is higher due to the high strength of Cherry rivets. Benefiting from larger rivet diameters or greater rivet quantities, the second peak load satisfies CL*m*2*d*4 < CL*m*2*d*4.8 < CL*m*3*d*4; Figure 10b illustrates the combination of low-strength GB rivet and high-strength Araldite adhesive. The first peak load is higher because of the superior strength of Araldite adhesive. The bonding area of the outermost layer for the three cases is 269.03 mm^2^, 420.36 mm^2^ and 377.83 mm^2^, respectively, leading to the relationship of the first peak load: GA*m*2*d*4 < GA*m*3*d*4 < GA*m*2*d*5. The second peak load is relatively low as a result of the low strength of GB rivets. Therefore, for material selection in adhesive–rivet hybrid repair, apart from ensuring that the performance of adhesive and rivet approximates the values calculated from Equation (12), the adoption of higher-strength rivets and adhesive allows the use of smaller rivet diameters. This further reduces the overall patch dimension and achieves structural weight reduction. The quantitative experimental results, including the first load (adhesive damage initiation load) and the second load (failure load), together with their corresponding displacements, are summarized in Table 9.

### 4.2. Structural Mechanical Characteristics Under Hybrid Adhesive–Rivet Interaction

#### 4.2.1. Rivet Load Characteristics Under Hybrid Adhesive–Rivet Interaction

**Figure 11:**
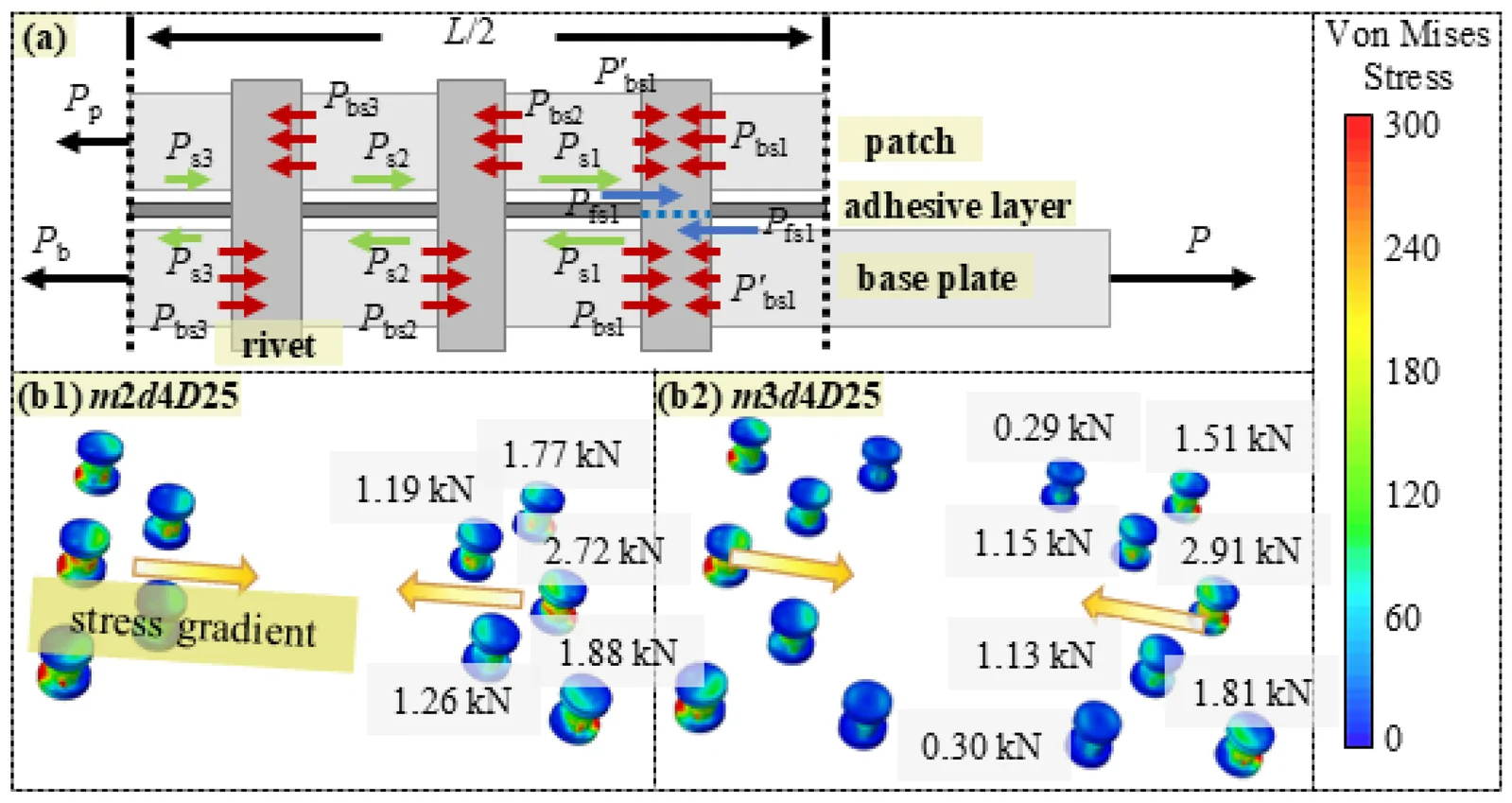
Force distribution of rivets in repaired specimen: (**a**) schematic of rivet force; (**b1**) rivet stress contour *m*2*d*4*D*25; (**b2**) rivet stress contour *m*3*d*4*D*25.

The mechanical behavior of the patched repaired structure is symmetric about the central axis, so only one side is selected for force analysis. Subjected to tensile load P, the base plate undergoes displacement. The outermost rivet hole of the base plate squeezes the lower portion of the outermost rivet and generates bearing force *P*_bs1_, driving the rivet to move along the tensile direction. The upper portion of the moving rivet squeezes the outermost rivet hole of the patch with an equal-magnitude force. Consequently, the patch moves together with the rivet in the tensile direction, and relative sliding between the patch and base plate induces shear deformation of the adhesive layer. Load transfer between the base plate and patch propagates sequentially from the outermost rivets and adhesive layer toward the central rivets of the repaired region. Shear force *P*_fs_ forms inside each rivet to resist the bearing force *P*_bs_ applied at the upper and lower ends, while the adhesive layer provides shear force *P*_s_ to restrain shear deformation. The relative displacement between the patch and base plate reaches the maximum at the outermost rivets, resulting in the maximum rivet shear force *P*_fs_ and adhesive shear force *P*_s_. The load carried by rivets decreases progressively toward the center of the repaired structure. The schematic diagram of load distribution among the rivet group is shown in Figure 11a. Hence, the outermost rivets are most prone to fracture.

The patch together with the upper half of rivets, and the base plate together with the lower half of rivets are selected as research objects, respectively. *P*_pi_ denotes the sectional force of the patch between the *i*-th and (*i* + 1)-th rows of rivets counting from the outermost side; *P*_bi_ represents the sectional force of the base plate between the *i*-th and (*i* + 1)-th rows of rivets counting from the outermost side.

(28) $$\left\{\begin{array}{l} P_{p1}=P_{fs1}+P_{s1}+P_{s0}\\ P=P_{b1}+P_{fs1}+P_{s1}+P_{s0} \end{array}\right.$$

For the second rivet from the outermost side:

(29) $$\left\{\begin{array}{l} P_{p2}=P_{fs2}+P_{fs1}+P_{s2}+P_{s1}+P_{s0}\\ P=P_{b2}+P_{\mathrm{fs}2}+P_{s2}+f_{2}+P_{s1}+P_{s0} \end{array}\right.$$

For the n-th rivet from the outermost side:

(30) $$\left\{\begin{array}{l} P_{\mathrm{pn}}=\sum_{i=1}^{n}P_{\mathrm{fsi}}+\sum_{i=0}^{n}P_{\mathrm{si}}\\ P=P_{\mathrm{bn}}+\sum_{i=1}^{n}P_{\mathrm{fsi}}+\sum_{i=0}^{n}P_{\mathrm{si}} \end{array}\right.$$

Analysis of the above equations reveals that the axial sectional force of the patch between adjacent rivet rows increases gradually from the outermost position toward the center, while that of the base plate decreases correspondingly. Since the base plate has larger dimensions than the patch, the axial displacement of the base plate exceeds that of the patch under identical load. Moreover, the displacement difference between the base plate and the patch becomes more significant toward their outer edges, inducing upward deflection of the patch. The outer rivets are subjected to larger shear force and bending moment simultaneously to accommodate the displacement mismatch between the base plate and patch. Consequently, the rivet load decreases gradually from the outer rivets to the central rivets. This finding agrees with the conclusion in Reference [23]. The maximum stress occurs in the outermost rivets, which can be observed from the rivet stress contour in Figure 11(b1,b2).

#### 4.2.2. Adhesive Layer Load Characteristics Under Hybrid Adhesive–Rivet Interaction

**Figure 12:**
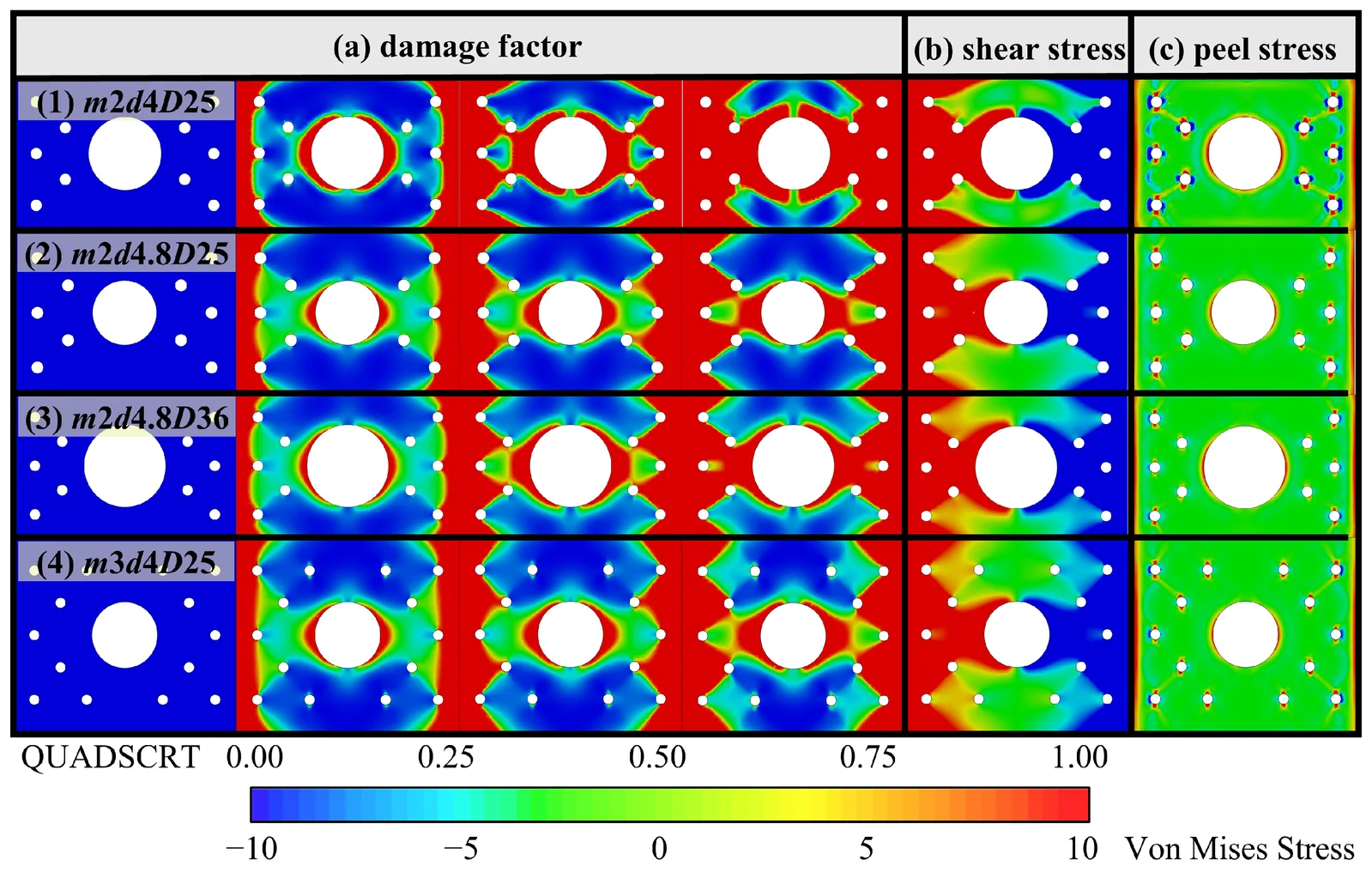
Mechanical characteristics of adhesive layer: (**a**) damage variable; (**b**) shear stress; (**c**) peel stress.

For mechanical (riveted) connections, load transfer between the base plate and patch follows a discrete stepped pattern. Loads are transferred from the base plate to the patch via bearing of discrete rivets according to stiffness distribution, thereby alleviating the load borne by the base plate. In contrast, the aerospace adhesive for adhesive bonding is uniformly distributed between the base plate and patch. The load carried by the base plate is continuously transmitted to the patch through the tangential shear force *P*_s_ of the adhesive layer. The relative displacement between the base plate and patch reaches the maximum at the outermost region, leading to the maximum strain and shear stress within the adhesive layer. Consequently, the outer adhesive layer fails first. The evolution of the adhesive layer damage variable is illustrated in Figure 12a.

Shear stress concentrates on both sides of the adhesive layer and the hole peripheries along the tensile direction. The shear stresses on the two sides act in opposite directions and are symmetric about the hole central axis, as shown in the shear stress contour in Figure 12b. High shear stress appears at the outermost region of the patch and the zone extending from the rivet hole array toward the circular hole along the tensile direction. By contrast, the shear stress around the circular hole perpendicular to the tensile direction remains low. The shear stress within the adhesive layer is governed by the relative displacement between the base plate and the patch. The region along the tensile direction near the circular hole undergoes severe deformation. Bending deformation induced by large displacement at both sides of the patch, together with the deformation of the circular hole under tensile load, jointly generates high shear stress zones along the tensile direction. The shear stress between rivet rows is determined by load transfer among the base plate, patch and rivets. The adhesive layer shares part of the shear load carried by rivets, which homogenizes the overall load distribution. Furthermore, the adhesive layer between rivet rows serves as the primary load transfer zone, where relatively high shear stress is observed.

The peel stress contour of the adhesive layer is presented in Figure 12c. The maximum peel stress occurs on both sides of the adhesive layer, further demonstrating that debonding initiates at these regions, i.e., the two sides of the patch debond first. This phenomenon originates from the displacement mismatch between the patch and base plate under tensile loading. The displacement difference reaches its maximum at the outer sides, and considerable relative displacement induces upward bending of the patch edges, leading to the highest peel stress on both sides. Meanwhile, the bearing action of rivets against rivet holes increases the relative displacement between the patch and base plate. The shear strength of the adhesive layer around the holes rises, the tendency for debonding between the patch and base plate intensifies, and the peel stress of the adhesive layer increases accordingly. High peel stress is observed on both sides of the circular hole along the tensile direction. As the hole elongates along the tensile direction and deforms from a circle into an ellipse under tension, severe deformation emerges around the hole edges aligned with the tensile direction, which promotes debonding with the patch and elevates the peel stress.

### 4.3. Failure Analysis of Hybrid Adhesive–Rivet Structures

#### 4.3.1. Stress Interference Around Holes in Hybrid Adhesive–Rivet Joints

**Figure 13:**
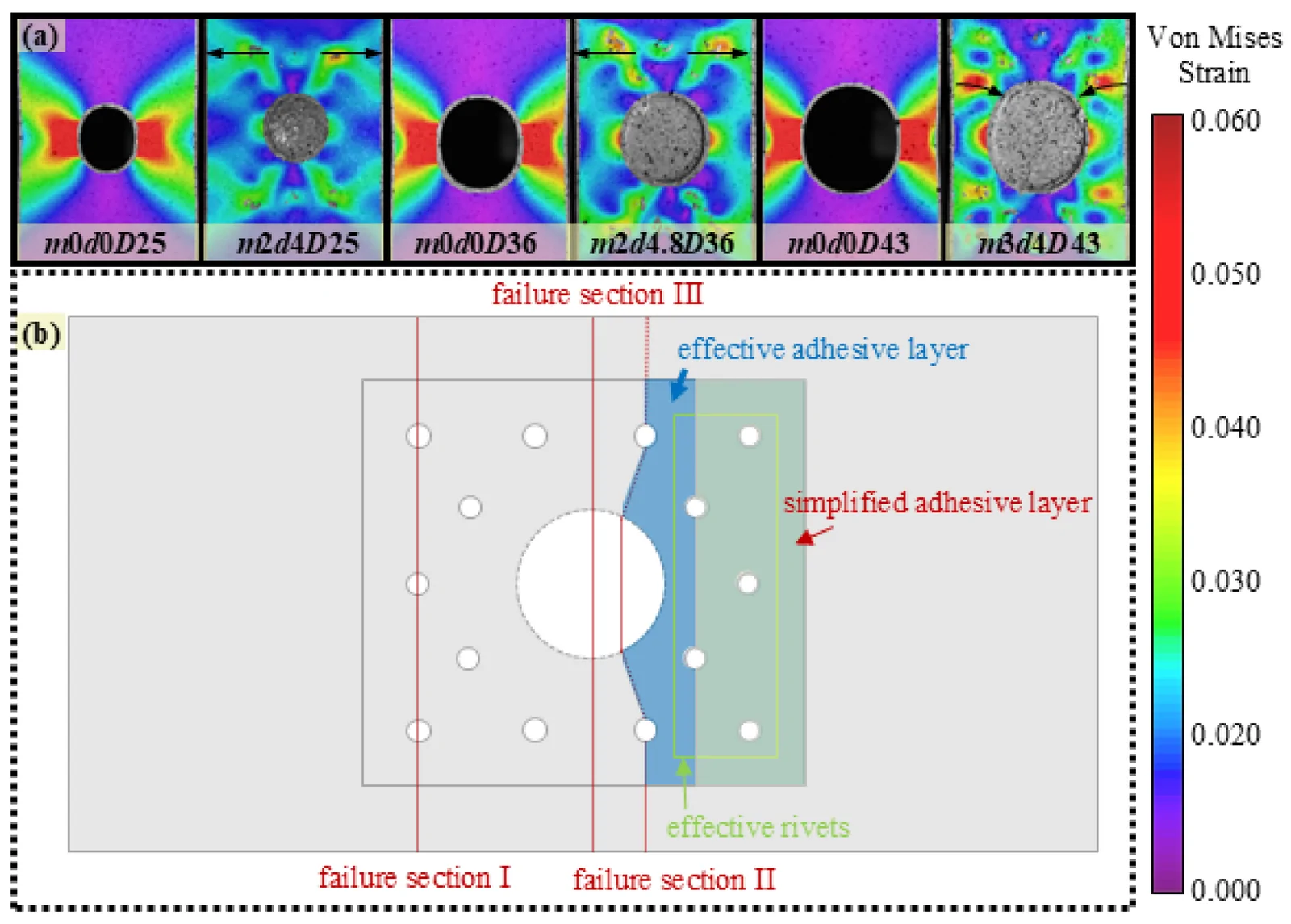
Contours and failure cross-section: (**a**) comparison of strain contours between repaired and intact specimens; (**b**) schematic of failure cross-section. The black arrows indicate the propagation cross-sections of structural fracture.

The DIC strain contours of hybrid adhesive-rivet repaired structures and unrepaired structures with various hole diameters are compared in Figure 13a. For unrepaired structures with different hole sizes, stress concentration emerges at the hole edges, and plastic zones propagate from the hole periphery toward the approximate 45° direction, forming an X-shaped distribution. As the hole diameter increases, the residual cross-sectional area decreases, and the remaining material bears higher external load, resulting in elevated stress levels and aggravated stress concentration. The corresponding failure mode is fracture across the cross-section containing the central hole. For hybrid adhesive–rivet repaired structures, initial stress concentration occurs at the outermost rivet hole closest to the edge of the base plate and then gradually extends toward inner rivet rows. Stress fields around the innermost rivet holes interact with the central circular hole, generating local stress concentration zones, while the stress magnitude on both sides of the central hole remains the lowest. In general, rivet holes introduce local stress concentration, yet the load transfer mechanism of hybrid adhesive–rivet joints alleviates the intensity of stress concentration. Compared with unrepaired structures, the repaired structures exhibit more uniform stress distribution, and the failure mode transforms into fracture originating from rivet holes.

#### 4.3.2. Failure Modes of Hybrid Adhesive–Rivet Structures

Only one half of the repaired structure contributes effectively to load bearing after hybrid adhesive–rivet repair. With rational rivet arrangement, the patch generally avoids fracture failure. The failure mode is governed by the combined effect of the location of critical sections and the effective hybrid adhesive–rivet interaction. Experimental and theoretical analyses identify three critical sections:

Section I: base plate section passing through the outermost row of rivet holes. Multiple rivet holes on this section interact with one another and induce severe mutual stress concentration. Load flow has to bypass the holes due to material discontinuity, leading to substantially elevated stress levels. The outermost rivet holes are closest to the edge of the base plate, forming structural weak zones. Furthermore, this section receives no supporting shear force from rivets, and only the edge margin of the patch serves as the effective adhesive area. According to the load-bearing Formula (34), this section is highly susceptible to failure.

Section II: the section where the innermost rivets interact with the central circular hole. If the central hole diameter is excessively large or the spacing between the circular hole and innermost rivet holes is insufficient, intensive stress interference occurs between the holes. The effective rivets include all rows except the innermost rivets, and the effective adhesive area covers the entire adhesive region from this failure section to the patch edge. This section possesses abundant effective rivets and adhesive area; thus, failure takes place only when the damaged hole is sufficiently large to produce strong coupling with adjacent rivet holes. Such failure mode can be suppressed by increasing the projected distance along the tensile direction between the innermost rivet holes and the central hole.

Section III: cross-section containing the central hole with the minimum net area. This section has the smallest cross-sectional area of the base plate, yet the entire half-side structure of rivets and adhesive layer participates in load transfer. Failure at this section requires an extremely large, damaged hole diameter. In practice, the central hole is relatively far from the base plate edge, so initial stress concentration hardly emerges here. This section is the safest among the three critical sections.

Consequently, the risk level of the three critical sections follows the order: Section I > Section II > Section III. The schematic of critical sections is shown in Figure 13b.

#### 4.3.3. Load Analysis upon Failure of Hybrid Adhesive–Rivet Structures

Combined with the mechanical characteristics of riveted and adhesive joints presented in Section 4.2, the mechanical behavior of structures repaired by hybrid adhesive-rivet joints is analyzed, as illustrated in Figure 11a. Subjected to external tensile load *P* applied on the base plate of the hybrid adhesive–rivet structure, both the adhesive layer and rivets participate in load transfer. The continuous load transfer of the adhesive layer reduces the gradient of load variation among rivets and achieves a more uniform stress distribution. Overall, the mechanical response bears similarities to fully riveted or fully bonded joints. The primary distinction lies in the replacement of frictional force between plates in riveted connections with shear force from the adhesive layer. Taking the cross-section passing through the base plate and patch, the sum of the load carried by the patch *P*_p_ and the load carried by the base plate Pb equals the external load *P*, which yields:

(31) $$P=P_{p}+P_{b}$$

For the patch, it is subjected to rivet bearing force Pbs and adhesive shear force *P*_s_. The rivet bearing force is equivalent to the internal shear force *P*_fs_ on the rivet cross-section, namely:

(32) $$P_{p}=P_{\mathrm{fs}}+P_{s}$$

Combining experimental results and theoretical calculations, rational rivet arrangement can prevent cross-sectional failure of the patch, and failure mainly occurs in the base plate. Substituting Equations (31) and (32), the externally applied load *P* at the failure of the base plate is expressed as:

(33) $$P=P_{b}+P_{\mathrm{fs}}+P_{s}$$

That is, the failure load of the hybrid adhesive–rivet repaired structure consists of the sum of the failure load for base plate cross-section fracture, rivet shear failure load and adhesive shear failure load, which serves as the foundation for strength evaluation of hybrid adhesive–rivet repaired structures.

For simplified expression, the rivet shear force *P*_fs_ is abbreviated as rivet force *P*_r_, and the adhesive shear force Ps is abbreviated as adhesive force *P*_a_. Accordingly, Equation (33) can be rewritten as:

(34) $$P=P_{b}+P_{r}+P_{a}$$

The loads carried by the adhesive layer and the rivets prior to adhesive debonding were extracted and are summarized in Table 10. The results indicate that, before adhesive failure, the external load is primarily carried by the adhesive layer, while the rivets have not yet reached their ultimate load-carrying capacity. After debonding initiates at the adhesive boundary, the load is progressively transferred to the rivets, which subsequently become the primary load-carrying component. Before adhesive failure, the maximum load share carried by the adhesive layer reaches 67.25%, with the corresponding rivet load share being 32.75%.

## 5. Strength Evaluation of Structures Repaired by Hybrid Adhesive–Rivet Joints

### 5.1. Variation Law of Structural Strength of Repaired Specimens Under Various Working Conditions

The failure loads obtained from experiments and numerical simulations are compared. Three repair schemes are adopted for specimens with a hole diameter of 25 mm: double-row rivets with a diameter of 4 mm, double-row rivets with a diameter of 4.8 mm, and triple-row rivets with a diameter of 4 mm. The failure loads of the three structures are approximate, while the failure load of Scheme 1 is slightly lower than that of Scheme 2, and Scheme 2 is slightly lower than Scheme 3. This is attributed to the fact that the patch dimension satisfies Scheme 3 > Scheme 2 > Scheme 1, which provides a larger effective adhesive area. The increased number of rivets enables more uniform load transfer between the base plate and patch and improves the load-bearing capacity. By comparing the failure loads of structures with identical repair schemes but different hole diameters, it can be found that the failure load of repaired structures decreases as the hole diameter increases. Comparison of strain contours for cases *m*2*d*4.8*D*25 and *m*2*d*4.8*D*36 in Figure 7 indicates that the strain around the hole for *D*36 is remarkably higher than that for *D*25. With equal rivet spacing, the distance between the edge of the *D*36 hole and the innermost rivets is shorter, generating distinct strain coupling zones. Overall, the strain level of the *D*36 model is higher than that of the *D*25 model.

### 5.2. Strength Evaluation Method for Repaired Structures

The rivet arrangement governs the failure mode of the base plate, and the failure load depends on the location of critical sections. Therefore, the key to evaluating the strength of repaired structures lies in identifying the correct critical section. Combined with the analysis in Section 4.3.2. According to the factors affecting the failure load of hybrid joints summarized in Section 4.3.3, the criterion for critical section selection is stated as follows: For a given cross-section, define the total load *P* as the sum of the base plate fracture load *P*_b_, the shear fracture load of rivets outside this section (excluding rivets on this section) *P*_r_, and the adhesive shear fracture load *P*_a_. The cross-section corresponding to the minimum value of *P* is identified as the critical section.

The failure load of the base plate at cross-section *i*, *P*_bi_, is expressed as

(35) $$P_{\mathrm{bi}}=\sigma_{b}A_{\mathrm{bi}}$$

where *σ*_b_ denotes the ultimate stress of the base plate, and *A*_bi_ is the effective cross-sectional area of the base plate.

The calculation formula for the effective cross-sectional area of the base plate at Section I is expressed as:

(36) $$A_{\mathrm{bI}}=\left(W-nd\right)\delta$$

where *W* denotes the width of the base plate, *n* is the number of outermost rivets, *d* represents the rivet diameter, and *δ* stands for the thickness of the base plate.

The simplified calculation formula for the effective cross-sectional area of the base plate at Section II is expressed as:

(37) $$A_{\mathrm{bII}}=\left(W-D-n_{i}d\right)\delta$$

where *D* is the hole diameter, *n*_i_ represents the number of innermost rivets.

The failure load of effective rivets for cross-section *i*, *P*_ri_, is given by:

(38) $$P_{\mathrm{ri}}=n_{\mathrm{ei}}\frac{\pi }{4}d^{2}\tau_{\mathrm{req}}$$

where *n*_ei_ is the number of effective rivets (rivets located on the cross-section are excluded); *d* represents the rivet diameter; *τ*_req_ is the equivalent shear strength of open-type rivets treated as solid rivets. For structural solid rivets, the equivalent shear strength equals the shear strength of the rivet material, namely *τ*_req_ = *τ*_r_.

The failure load of the effective adhesive layer for cross-section *i*, *P*_ai_, is written as:

(39) $$P_{\mathrm{ai}}=\tau_{a}A_{\mathrm{ai}}$$

where *τ*_a_ is the shear strength of the adhesive layer, and *A*_ai_ represents the effective adhesive area, which refers to the net adhesive area from the failure cross-section to the nearest patch edge, namely the total area excluding the regions occupied by rivet holes and the central circular hole.

For Section I, the expression for the effective cross-section of the adhesive layer is:

(40) $$A_{\mathrm{aI}}=cnt-\frac{n}{2}\frac{\pi }{4}d^{2}$$

For Section II, as analyzed in Section 4.2.2, high shear stress of the adhesive layer concentrates between rivet rows and on both sides of holes along the tensile direction. The effective adhesive area can be simply equivalent to the net adhesive area between the outermost two rivet rows and the patch edge (with the area of rivet holes deducted), expressed as:

(41) $$A_{\mathrm{aII}}=\left(a+c\right)nt-n_{\mathrm{eII}}\frac{\pi }{4}d^{2}$$

The total failure load *P*_i_ for cross-section *i* (*i* = I, II) is:

(42) $$P_{i}=\left(P_{\mathrm{bi}}+P_{\mathrm{ri}}+P_{\mathrm{ai}}\right)\beta$$

where *β* denotes the reduction coefficient. The above evaluation assumes that the failure cross-section, rivets and adhesive layer all reach their ultimate strength simultaneously upon failure. In practice, at least one of the three load components cannot attain the ultimate strength due to the matching interaction between adhesive and rivets, as well as the modulus mismatch among the adhesive, rivets and base plate. To simplify the evaluation method, the internal load transfer path within the structure is neglected, and the reduction coefficient *β* is introduced.

The ultimate failure load *P* of the structure is:

(43) $$P=\min P_{i}$$

where the evaluated critical section corresponds to the cross-section with the minimum failure load *P*.

**Figure 14:**
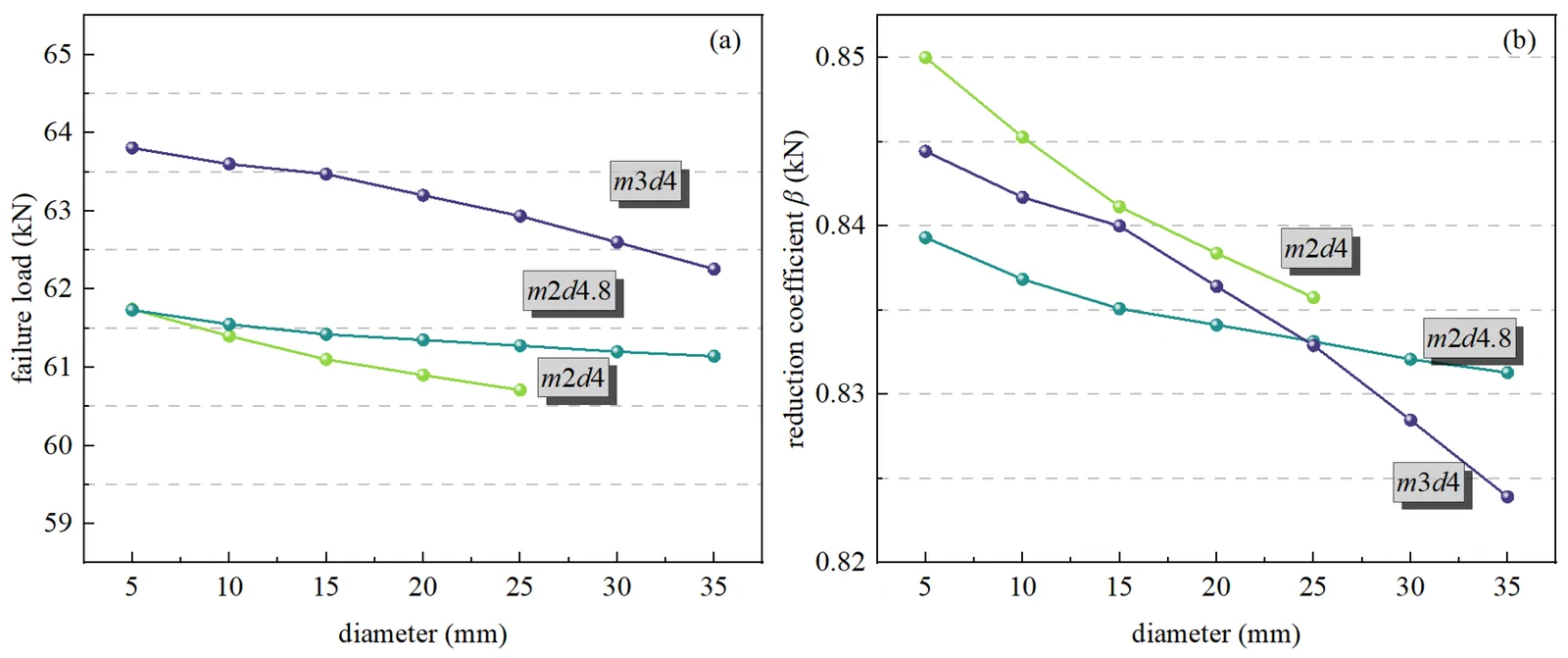
Finite element results of the repaired specimens: (**a**) failure load; (**b**) reduction coefficient.

The finite element simulations were further extended to 20 configurations of *m*2*d*4, *m*2*d*4.8, *m*3*d*4 and with different damage-hole diameters ranging from 5 to 35 mm at 5 mm intervals, as shown in Figure 14.

In the existing literature [22], the reduction coefficient *β* is set to 0.88. Nevertheless, practical calculation needs to account for multiple factors including the matching performance of adhesive–rivet joints, premature structural failure induced by rivet holes, the incapability of adhesives and rivets to reach their ultimate strength, and the coupled bending-shear loading state of hybrid joints. Accordingly, *β* is adopted as 0.82 in this work. Considering the 2–5% discrepancy between the experimental and numerical results, an additional 2% reduction was introduced. Consequently, the reduction coefficient *β* was set to 0.80. The coefficient was also rounded to facilitate simplified engineering calculations. The resulting assessed failure loads are compared with the investigated experimental dataset in Table 11. Because the reduction coefficient includes an empirical correction partly informed by the experiment–simulation discrepancy, this comparison quantifies agreement with the investigated dataset and should not be interpreted as fully independent predictive validation.

For the adhesive–rivet hybrid structure, failure Section I corresponds to the cross-section through the outermost rivet row. Once this section fails, the material outside the critical section fractures and separates from the remaining structure. Consequently, the rivets located on this section can no longer provide effective constraint to the detached outer portion, and the rivet contribution to the load-carrying capacity of Section I is therefore taken as zero.

Within the investigated experimental dataset, the assessed failure loads differ from the experimental values by at most 6.37%, with the largest deviation occurring for the Section II failure mode. Because the reduction coefficient contains an empirical correction partly informed by the experiment–simulation discrepancy, this result should be interpreted as agreement with the investigated dataset rather than as fully independent predictive validation. The assessment of Section II is more complex because the rivet load decreases progressively from the outer rows toward the inner rows, which may lead to an overestimation of the effective rivet contribution in the current simplified model. Further refinement of the rivet load-distribution treatment could therefore improve the assessment for this failure mode. Nevertheless, the comparison indicates reasonable agreement across the configurations investigated in this study, while independent experiments are required to evaluate broader predictive applicability.

### 5.3. Evaluation Method of Repair Effect

The repair effectiveness should be evaluated from two aspects: the additional mass introduced by the repair and the corresponding increase in structural load-carrying capacity. A repair scheme with lower added mass and greater strength enhancement is considered more efficient. Therefore, a new parameter, termed the repair rate *R*, is introduced in this study and defined as the ratio of the strength increment after repair, Δ*P*, to the additional structural mass, Δ*m*. This parameter characterizes the overall performance of the repair scheme. A higher repair rate *R* indicates greater reinforced strength or lower weight increment, which represents superior repair effectiveness. The expression is written as:

(44) $$R=\frac{\Delta P}{\Delta m}$$

Δ*P* is defined as the strength of the repaired structure *P* minus the residual strength of the unrepaired structure *P*_d_.

(45) $$\Delta P=P-P_{d}$$

The residual strength of the unrepaired structure *P*_0_ can be calculated using the $\Delta P=P-P_{d}P$ evaluation method proposed in Reference [23]:

(46) $$P_{d}=\sigma_{b}\left(W_{b}-D\right)\delta \beta$$

where *W*_b_ is the width of the base plate, *D* denotes the diameter of the central circular hole, and *t* represents the thickness of the base plate.

Δ*m* stands for the additional mass introduced by repair, including the mass of the patch *m*_p_, the mass of rivets *m*_r_, and the mass of the adhesive layer *m*_a_.

(47) $$\Delta m=m_{p}+m_{r}+m_{a}$$

Taking the *m*2*d*4*D*25 configuration as an example, the patch dimensions are mm, the adhesive-layer thickness is 0.1 mm, and the adhesive density is 1.4 g/cm^3^. The aluminum alloy patch has a thickness of 2.0 mm and a density of 2.7 g/cm^3^. After excluding the rivet holes, the calculated patch mass is 21.28 g; whereas, the adhesive mass is only 0.55 g, accounting for 2.51% of the total added mass. Therefore, the adhesive mass can be neglected in the simplified mass evaluation. In addition, aerospace rivets are generally made of lightweight aluminum alloys, and the rivet mass approximately compensates for the material removed by the rivet holes in the patch. Since the patch thickness is also much greater than the adhesive-layer thickness, the additional repair mass, Δ*m*, can be approximately taken as the mass of the intact patch without rivet holes.

(48) $$\Delta m=\rho_{p}W_{p}L_{p}t$$

where *ρ*_p_ is the density of the patch, *W*_p_ is the patch width, *L*_p_ is the patch length, and t denotes the patch thickness.

The structural strength recovery ratio *r* is defined as the ratio of the strength of the repaired structure *P* to the strength of the intact structure *P*_0_, namely:

(49) $$r=\frac{P}{P_{0}}$$

The structural strength strengthening ratio *s* represents the ratio of the strength increment (*P* − *P*_d_) to the strength of the damaged structure *P*_d_, expressed as:

(50) $$s=\frac{P-P_{d}}{P_{d}}$$

From experimental results, the failure loads of unrepaired plates with central hole diameters of 25 mm, 36 mm and 43 mm are 50.13 kN, 41.19 kN and 34.83 kN, respectively, and the strength of the intact specimen is 72.75 kN. The repair rate, recovery ratio and strengthening ratio under various working conditions are calculated via the above method, and the results are summarized in Table 12.

Under the above working conditions, the recovery ratio of structures with hybrid adhesive–rivet repair exceeds 74% in all cases. As the central hole diameter increases, the strengthening ratio of repaired structures rises accordingly. Meanwhile, the repair rate increases when the hole size approaches the limit hole dimension applicable to the corresponding repair scheme, which can be observed from cases *m*2*d*4*D*25, *m*2*d*4.8*D*36 and *m*3*d*4*D*43. For structures with identical central hole sizes, repair schemes adopting smaller rivet diameters and fewer rivet rows yield higher repair rates and superior repair effectiveness, as demonstrated by cases *m*2*d*4*D*25, *m*2*d*4.8*D*25 and *m*3*d*4*D*25.

Merah et al. [25] conducted adhesive-bonded, riveted, and adhesive–rivet hybrid repair tests on AL2024-T3 aluminum alloy plates containing a 10 mm diameter circular hole. Their results showed that the circular hole reduced the structural strength by approximately 30%, while the adhesive–rivet hybrid repair increased the load-carrying capacity of the damaged plate by approximately 20%, corresponding to an estimated strength recovery level of approximately 84%. In the present study, the strength recovery ratios of the six repair configurations range from 74.46% to 83.58%, which are comparable to the results reported by Merah et al. Considering that larger damage-hole diameters of 25–43 mm were investigated in this study, the relatively high strength recovery levels demonstrate the effectiveness of the proposed adhesive–rivet strength-matching method for repairing aluminum alloy plates with relatively large perforation damage.

## 6. Conclusions

This study addresses the parameter design and residual-strength assessment of adhesive–rivet hybrid repairs for perforated aluminum alloy plates. A combined approach of theoretical analysis, quasi-static tensile testing, and finite element simulation was employed to investigate adhesive–rivet strength matching, load sharing, and structural failure behavior, and a residual-strength assessment method for the repaired structure was established. The main conclusions are as follows:

1. A quantitative parameter-matching method for adhesive–rivet hybrid repair was established. Based on the equal-strength and stiffness-coordination principles, calculation relationships for rivet diameter, pitch, edge distance, row spacing, and rivet number were developed. The shear load-carrying capacity of the adhesive layer was further matched with the strengths of the rivets and base plate, enabling the repair parameters to be determined from the damage-hole size and material load-carrying capacities. This provides a methodological basis for shifting repair design from empirical selection toward quantitative design.
2. The cooperative load-carrying behavior of the adhesive layer and rivets, as well as the main failure characteristics, was clarified. Numerical results show that, before significant adhesive damage, the adhesive layer carries a substantial proportion of the transferred load, with a maximum load share of 67.25%, while the corresponding rivet load share is 32.75%. This demonstrates that the adhesive layer can effectively reduce the load carried by the rivet group. The rivet loads generally decrease from the outer rows toward the inner rows, with the outermost rivet-hole region experiencing the highest local load. The repaired perforated plates mainly fail along the cross-section through the outermost rivet row; whereas, under certain geometric conditions the critical section may shift to the coupled region between the inner rivets and the damage hole.
3. A residual-strength assessment model considering different critical sections was developed. Based on the combined contributions of the effective base-plate section, effective rivet shear capacity, and effective adhesive load-carrying capacity, a residual-strength calculation method covering multiple failure modes was established. Within the range of specimens and parameters investigated in this study, the maximum deviation between the model predictions and experimental results is 6.37%, indicating that the proposed method can reasonably capture the strength variation associated with different damage sizes and rivet configurations. However, because the reduction coefficient in the model contains an empirical correction component, its broader applicability requires further validation through independent experiments.
4. The repair effectiveness of different adhesive–rivet configurations was quantitatively evaluated. The strength recovery ratios of the six perforated-plate repair configurations range from 74.46% to 83.58%, and the maximum strengthening ratio reaches 55.49%. For the same damage-hole size, configurations with smaller-diameter rivets and fewer rivet rows exhibit higher values of the proposed repair ratio. This indicates that appropriate adhesive–rivet parameter matching can restore structural load-carrying capacity while limiting additional repair mass, thereby providing a quantitative basis for comparing and selecting repair schemes for perforated aluminum alloy thin plates.

Overall, the principal contribution of this study is the establishment of a quantitative analysis framework linking damage size–adhesive–rivet parameter matching–critical-section identification–residual-strength assessment. The proposed framework can provide a reference for repair-parameter design and strength evaluation of perforation-damaged aerospace aluminum alloy thin-walled structures under fully cured conditions.

It should be noted that the present study is limited to 2 mm-thick 2A12-T4 aluminum alloy plates subjected to quasi-static tensile loading at room temperature under fully cured conditions. In addition, the perforated-plate repair tests mainly employed the Cherry CR3212 rivet and Araldite-2015 adhesive combination. Therefore, further validation is required before extending the conclusions to other plate thicknesses, material systems, adhesives, or joining configurations. Future work will focus on fatigue and impact loading, environmental aging of adhesives, different curing states including partially cured conditions, and optimization of adhesive-layer thickness. More extensive independent experiments and reliability analyses will also be conducted to further evaluate the applicability of the proposed strength-matching method and residual-strength model.

## Figures and Tables

**Table 1:** Butt joint specimens.

Specimen No.	Rivet Rows (One Side) (m)	Rivet Diameter (d)/mm	Rivet Pitch (t)/mm	Edge Margin (c)/mm	Row Pitch (a)/mm	Material
GA*m*2*d*4	2	4	17.6	8	10	GB + Araldite
GA*m*2*d*5	2	5	22	10	12.5	GB + Araldite
GA*m*3*d*4	3	4	24.4	8	10	GB + Araldite
CL*m*2*d*4	2	4	17.6	8	10	Cherry + Lord
CL*m*2*d*4.8	2	4.8	21.2	9.6	12	Cherry + Lord
CL*m*3*d*4	3	4	24.4	8	10	Cherry + Lord

**Table 2:** Hybrid adhesive–rivet repaired specimens.

Specimen No.	Rivet Rows (One Side) (m)	Rivet Diameter (d)/mm	Rivet Pitch (t)/mm	Edge Margin (c)/mm	Row Pitch (a)/mm	Rivet Quantity (One Side) (N)	Hole Diameter (D)/mm
*m*2*d*4*D*25	2	4	17.6	8	10	5	25
*m*2*d*4.8*D*25	2	4.8	21.2	9.6	12	5	25
*m*2*d*4.8*D*36	2	4.8	21.2	9.6	12	5	36
*m*3*d*4*D*25	3	4	24.4	8	10	7	25
*m*3*d*4*D*36	3	4	24.4	8	10	7	36
*m*3*d*4*D*43	3	4	24.4	8	10	7	43
*m*0*d*0*D*25	-	-	-	-	-	-	25
*m*0*d*0*D*36	-	-	-	-	-	-	36
*m*0*d*0*D*43	-	-	-	-	-	-	43

**Table 3:** Material properties of 2A12-T4 aluminum alloy [22,23] and CR3212 rivet [24].

Material	Elastic Modulus (*E*)	Poisson’s Ratio (*ν*)	Failure Strain (*ε*_f_)	Tensile Strength (*σ*_b_)	Shear Strength (*τ*_b_)
2A12-T4	69 GPa	0.33	0.19	481 MPa	305 MPa
Cherry CR3212	69 GPa	0.33	0.144	-	345 MPa

**Table 4:** Material properties of Araldite-2015 [15].

Material Parameter	Value
Elastic Modulus (*E*)	1.85 GPa
Shear Modulus (*G*)	0.56 GPa
Poisson’s Ratio (*ν*)	0.33
Maximum Normal Traction (*t*_n_^0^)	21.63 MPa
Maximum Shear Traction (*t*_s_^0^, *t_t_*^0^)	17.90 MPa
Mode I Fracture Energy (*G*_I_^C^)	0.43 N/mm
Mode II Fracture Energy (*G*_II_^C^)	4.70 N/mm

**Table 5:** Cohesive zone model mesh convergence.

Mesh Size/mm	Shear Stress/MPa	Peel Traction/MPa	PEEQ	Residual Bonded-Area Ratio
0.5 mm	17.89	21.47	0.054	0.743
1.0 mm	17.88	21.13	0.055	0.739
1.5 mm	17.87	20.37	0.069	0.728

**Table 6:** Rivet loads.

Mesh Size/mm	Minimum Load/kN	Maximum Load/kN	Total Load/kN
0.5 mm	1.27	2.78	8.11
1.0 mm	1.19	2.72	8.84
1.5 mm	1.95	3.86	13.37

**Table 7:** Numerical results of adhesive material sensitivity analysis.

Material Parameter	Adhesive Damage Initiation Load (*P*_i_)	Maximum Failure Load (*P*_u_)	Failure Displacement (*δ*_f_)	Residual Bonded-Area Ratio (*A*)
Baseline	60.44	62.02	5.59	0.739
0.8 *t*_n_^0^	60.77	62.10	5.64	0.735
1.2 *t*_n_^0^	60.31	62.01	5.60	0.710
0.8 *t*_s_^0^	60.28	61.97	5.64	0.784
1.2 *t*_s_^0^	60.64	62.16	5.64	0.730
0.8 *G*_I_^C^	60.42	62.02	5.55	0.718
1.2 *G*_I_^C^	60.47	62.00	5.59	0.731
0.8 *G*_II_^C^	58.77	61.47	5.30	0.630
1.2 *G*_II_^C^	61.82	62.47	5.84	0.732
0.8 *η*	60.78	62.05	5.64	0.747
1.2 *η*	60.19	62.02	5.54	0.710

**Table 8:** Normalized sensitivity coefficients of adhesive material parameters.

Material Parameter	*SP*_i_	*SP*_u_	*Sδ*_f_	*SA*
*t*_n_^0^	−0.019	−0.004	−0.021	−0.085
*t*_s_^0^	0.015	0.008	0	−0.182
*G*_I_^C^	0.002	−0.001	0.021	0.042
*G*_II_^C^	0.126	0.04	0.242	0.345
*η*	−0.024	−0.001	0.044	−0.122

**Table 9:** Experimental results of joint specimens.

Specimen No.	Adhesive Damage Initiation Load/kN	Displacement at Adhesive Damage Initiation/mm	Failure Load/kN	Displacement at Failure/mm
CL*m*2*d*4	10.25	0.30	12.80	1.15
CL*m*2*d*4.8	13.63	0.41	17.17	1.53
CL*m*3*d*4	17.58	0.47	21.49	0.80
GA*m*2*d*4	9.00	0.30	8.23	1.69
GA*m*2*d5*	14.07	0.51	8.94	1.92
GA*m*3*d*4	16.62	0.47	11.55	1.96

**Table 10:** Experimental results of hybrid adhesive–rivet repaired specimens.

Specimen No.	Maximum Adhesive Load/kN	Corresponding Total Rivet Load/kN	Adhesive Load Share	Rivet Load Share
*m*2*d*4*D*25	12.55	8.84	58.65%	41.34%
*m*2*d*4.8*D*25	15.27	7.43	67.25%	32.75%
*m*2*d*4.8*D*36	18.57	9.63	65.83%	34.17%
*m*3*d*4*D*25	17.89	9.14	66.18%	33.82%
*m*3*d*4*D*36	19.66	10.93	64.26%	35.74%
*m*3*d*4*D*43	21.59	12.14	64.00%	36.00%

**Table 11:** Comparison of residual-strength assessment results with the investigated experimental dataset.

Specimen No.	Adhesive Load/kN	Rivet Load/kN	Base Plate Load/kN	Evaluated Failure Load/kN	Experimental Failure Load/kN	Error/%	Failure Section
*m*2*d*4*D*25	7.22	0	65.41	58.11	59.59	−2.48	I
*m*2*d*4.8*D*25	10.44	0	63.10	58.84	60.23	−2.31	I
*m*2*d*4.8*D*36	10.44	0	63.10	58.84	57.59	2.15	I
*m*3*d*4*D*25	10.14	0	65.41	60.44	60.80	−0.59	I
*m*3*d*4*D*36	10.14	0	65.41	60.44	58.75	2.80	I
*m*3*d*4*D*43	22.46	21.67	27.89	57.62	54.17	6.37	II

**Table 12:** Evaluation of repair effect under various working conditions.

Specimen No.	Experimental Failure Load/kN	Recovery Ratio (*r*)/%	Strengthening Ratio (*s*)/%	Repair Rate (*R*)/kN/g
*m*2*d*4*D*25	59.59	81.91	18.86	0.43
*m*2*d*4.8*D*25	60.23	82.79	20.14	0.33
*m*2*d*4.8*D*36	57.59	79.17	39.80	0.48
*m*3*d*4*D*25	60.80	83.58	21.29	0.31
*m*3*d*4*D*36	58.75	80.76	42.61	0.46
*m*3*d*4*D*43	54.17	74.46	55.49	0.47

## Data Availability

Data is contained within the article.

## Nomenclature

**Symbol**	**Definition**	**Unit**
*P*	Failure load of the repaired structure	kN
*P*_0_	Failure load of the intact structure	kN
*P*_d_	Residual failure load of the unrepaired damaged structure	kN
*P*_b_	Load carried by the base plate	kN
*P*_p_	Load carried by the patch	kN
*P*_r_	Load carried by the rivets	kN
*P*_a_	Load carried by the adhesive layer	kN
*P*_i_	Predicted failure load of the (i)-th critical section	kN
*P*_ri_	Load carried by the effective rivets at the (i)-th critical section	kN
*P*_ai_	Load carried by the effective adhesive layer at the (i)-th critical section	kN
*P*_bi_	Load carried by the base plate at the (i)-th critical section	kN
*P*_fs_	Rivet shear force	kN
*P*_s_	Adhesive-layer shear force	kN
*P*_bs_	Bearing force between the rivet and rivet hole	kN
*D*	Diameter of the perforation damage	mm
*d*	Rivet diameter	mm
*t*	Rivet pitch	mm
*a*	Row spacing of rivets	mm
*c*	Rivet edge distance	mm
*m*	Number of rivet rows on one side of the patch	–
*n*	Number of rivets in the outermost row	–
*N*	Total number of rivets on one side of the patch	–
*n*_ei_	Number of effective rivets at the (i)-th critical section	–
*W*_b_	Width of the base plate	mm
*W*_p_	Width of the patch	mm
*L*_p_	Length of the patch	mm
*δ*	Thickness of the base plate/patch	mm
*A*_ai_	Effective adhesive area at the (i)-th critical section	mm^2^
*σ*_b_	Ultimate tensile strength of the base plate	MPa
*τ*_r_	Shear strength of the rivet material	MPa
*τ*_req_	Equivalent shear strength of the rivet	MPa
*τ*_a_	Shear strength of the adhesive layer	MPa
*τ*_raeq_	Equivalent shear strength of the adhesive–rivet hybrid connection	MPa
*β*	Reduction coefficient in the residual-strength model	–
*f*	Safety factor for repair design	–
*t*_n_^0^	Nominal normal traction at damage initiation	MPa
*t*_s_^0^	Nominal shear traction at damage initiation	MPa
*G*_I_^C^	Critical Mode-I fracture energy	N/mm
*G*_II_^C^	Critical Mode-II fracture energy	N/mm
*η*	Benzeggagh–Kenane mixed-mode parameter	–
Δ*P*	Increase in load-carrying capacity after repair	kN
Δ*m*	Additional mass introduced by the repair	g
*m*_p_	Mass of the patch	g
*m*_r_	Mass of the rivets	g
*m*_a_	Mass of the adhesive layer	g
*ρ*_p_	Density of the patch material	g/cm^3^
*r*	Strength recovery ratio	%
*s*	Strengthening ratio	%
*R*	Proposed repair ratio	kN/g
